# Electroactive Proteinoid–Quantum Dot Systems

**DOI:** 10.1002/smsc.202500418

**Published:** 2025-10-26

**Authors:** Panagiotis Mougkogiannis, Andrew Adamatzky

**Affiliations:** ^1^ Unconventional Computing Laboratory University of the West of England Bristol BS16 1QY UK

**Keywords:** proteinoids, quantum dots, self‐assembly, synaptic plasticity

## Abstract

Proteinoid–quantum dot (QD) conjugates are a new class of bioquantum hybrid materials combining biological self‐assembly with semiconductor nanocrystal electronic properties. This study describes the synthesis and analysis of Glu‐Phe‐Asp‐Cys proteinoid–QD networks using sulfosuccinimidyl 4‐(N‐maleimidomethyl)cyclohexane‐1‐carboxylate (sulfo‐SMCC) cross‐linking chemistry, achieving 80–90% conjugation efficiency. Scanning electron microscopy reveals a morphological transformation from spherical precursors to toroidal nanostructures with outer diameters of 145.2±18.7 nm and central cavities of 102.3±15.2 nm. The hybrid networks exhibit spontaneous electrochemical oscillations (0.03 to 0.11 Hz, 297‐485  mV) reproducible across trials. QD incorporation enhances signal amplitude 41‐fold (1999  mV vs. 48.8  mV) via surface plasmon coupling. Optimal charge transfer resistance for biosensing is ≈5250  Ω. Electron transfer kinetics follow first‐order decay ($\alpha =0.0032\;\;{\text{Hz}}^{-1}$). The networks respond to structured binary input over 5 days, displaying frequency synchronization at f=0.022217  Hz. Magnitude‐squared coherence values are 0.90 for pure proteinoids and 0.85 for conjugates. The system exhibits adaptive response‐like behavior through structural transformations, enabling applications in neuromorphic computing, adaptive biosensors, and information processing architectures.

## Graphical abstract

Proteinoid‐quantum dot conjugates form toroidal nanostructures (145.2 nm outer diameter, 102.3 nm cavity) via glutamic acid‐phenylalanine‐aspartic acid‐cysteine cross‐linking with sulfo‐SMCC (sulfosuccinimidyl 4‐(N‐maleimidomethyl)cyclohexane‐1‐carboxylate). These bioquantum hybrids exhibit spontaneous electrochemical oscillations (0.03‐0.11 Hz, 297‐485 mV) with 41‐fold quantum dot signal enhancement. The system demonstrates adaptive response‐like behavior through frequency synchronization and structural transformations, enabling applications in neuromorphic computing and adaptive biosensing with optimal charge transfer resistance of approximately 5250 Ω (ohms).

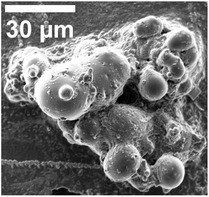

## 1 Introduction

Proteinoids are thermal protein‐like polymers that can self‐assemble into microspheres and complex structures. This assembly is driven by hydrophobic interactions, electrostatic stabilization, and hydrogen bonding. Fox and Harada first synthesized these materials through thermal copolymerization of amino acids at 170 °C. Despite being synthetic, proteinoids display remarkable protein‐like properties.^[^ ^1^, ^2^, ^3^ ^]^ Chromatographic analysis showed that all common amino acids were present in the resulting material, with the exception of tryptophan.^[^ ^4^ ^]^ A molar excess of dicarboxylic amino acids—particularly glutamic and aspartic acid—is essential for efficient thermal synthesis. This compositional requirement facilitates proper polymer formation, often yielding more than 15% by weight.^[^ ^5^, ^6^, ^7^ ^]^ Proteinoids possess unique features that distinguish them from conventional synthetic polymers. They spontaneously form microspheres in aqueous environments, exhibit primitive enzyme‐like activities, and display assembly patterns reminiscent of biological membranes. Proteinoids are amphiphilic, containing hydrophobic, hydrophilic, and charged amino acids. This amphiphilicity enables them to self‐organize into complex 3D structures suitable for bioconjugation and hybrid material fabrication. Their natural compatibility, flexible backbone, and molecular recognition capabilities make proteinoids well‐suited for integration with quantum systems.^[^ ^8^, ^9^, ^10^ ^]^ By combining biological self‐assembly with quantum properties, these materials hold promise for the development of novel computing platforms.^[^ ^4^, ^11^, ^12^, ^13^, ^14^, ^15^ ^]^ In this study, we focus on the experimental synthesis and characterization of Glu‐Phe‐Asp‐Cys proteinoid–QD conjugates, demonstrating high conjugation efficiency, unique toroidal morphologies, and enhanced electrochemical oscillations as key findings.

Quantum dots (QDs) are tiny semiconductor crystals. Their optical and electronic properties change with size. This tunable photoluminescence makes QDs useful for bioconjugation and signal amplification in hybrid nanomaterials. These engineered nanomaterials have great optoelectronic properties.^[^ ^16^, ^17^, ^18^, ^19^, ^20^ ^]^ This is because quantum confinement affects how charge carriers behave in three dimensions. It creates distinct energy levels. Also, it allows for size‐tunable emission wavelengths that cover the visible to near‐infrared spectrum. The surface chemistry of QDs is key to their function.^[^ ^21^, ^22^, ^23^, ^24^, ^25^ ^]^ Colloidal QDs are often capped with organic or inorganic ligands. These ligands help prevent clumping and create reactive sites. This allows targeting biomolecules to attach, which helps integrate QDs into complex nanoplatforms for biomedical uses.^[^ ^26^, ^27^, ^28^ ^]^ New core‐multishell designs have reached nearly 100% in photoluminescence quantum yields. This is thanks to better charge carrier localization. They also show great photostability and broad extinction coefficients, which beat standard fluorescent dyes.^[^ ^29^, ^30^, ^31^, ^32^ ^]^ QDs have special electronic features beyond their optical properties. They show metal‐enhanced photoluminescence through charge exchange. This process creates hot holes that can change emission intensity. It does this by turning charged non‐emitting states back into neutral emitting states. Quantum dots (QDs) have tunable optical properties and strong bioconjugation chemistry. They also exhibit quantum mechanical charge transfer. So, QDs are great for making hybrid bioquantum systems. These systems use semiconductor nanocrystals to boost and control biological processes at the molecular level.^[^ ^26^, ^32^, ^33^, ^34^ ^]^ The conjugation of proteinoids with quantum dots uses thiol‐maleimide chemistry.^[^ ^35^, ^36^, ^37^, ^38^, ^39^ ^]^ This creates nanocomposites. They mix the self‐organizing traits of biological polymers with the quantum effects found in semiconductor nanostructures. This bioconjugation strategy uses the maleimide‐thiol coupling reaction. This reaction happens through Michael addition and creates stable thioether linkages. It works well in mild aqueous conditions, which helps keep the structure of protein‐like polymers and quantum dot surfaces intact. This conjugation method is versatile and works in many systems. For example, it helps create multifunctional nanogels that use heat to assemble and link for drug delivery and imaging. It also applies to peptoid‐quantum dot assemblies, where special capping ligands bond to control surface density and organization. Recent advances show that maleimide‐thiol conjugation can connect biomolecules to quantum defect‐functionalized carbon nanotubes and other nanomaterials.^[^ ^40^, ^41^, ^42^ ^]^ This reaction acts as an anchor while keeping optical properties intact and adding new functionalities. Some worry about thiosuccinimide link stability because of retro‐Michael reactions or thiol exchange.^[^ ^43^, ^44^, ^45^ ^]^ Yet, applying controlled force can strengthen these bonds. This means that using mechanical processing while assembling proteinoids and quantum dots may actually make the conjugates more stable. Combining cysteine‐containing proteinoids with maleimide‐functionalized quantum dots is a strong bioconjugation strategy. It retains the self‐assembly traits of protein‐like polymers. It also keeps the unique optoelectronic properties of semiconductor nanocrystals. This approach allows for hybrid bioquantum systems that can sense and compute in new ways.^[^ ^40^, ^44^, ^46^, ^47^ ^]^ Our experiments reveal spontaneous voltage oscillations with frequencies of 0.03–0.11 Hz and amplitudes of 297–485 mV, reproducible across trials, with QD incorporation amplifying signals by a factor of 41.

Unconventional computing uses nontraditional materials for processing information.^[^ ^48^ ^]^ Biological and quantum systems have unique benefits. They excel in parallel processing, adapt easily, and are more energy‐efficient than silicon‐based systems.^[^ ^49^, ^50^, ^51^, ^52^, ^53^ ^]^ Unconventional computing differs from regular digital computers. Instead of using discrete elements, it takes advantage of the natural properties of materials. This allows for *in materia* computation. Here, networks of tiny elements create collective phenomena and complex behaviors. These features offer processing abilities like those of the brain. These approaches consist of neuromorphic systems, which use self‐assembled nanostructures that exhibit spatiotemporal correlations and synaptic connectivity; photonic neural networks, which rely on light–matter interactions for fast and energy‐efficient computing; memristive crossbar arrays, which enable in‐memory computing and help to overcome the von Neumann bottleneck; and design‐less networks, which operate at criticality and leverage avalanche effects. They offer new choices that go beyond traditional architectures. For example, the receptron device made from nanostructured gold films can classify complex tasks. It does this by reorganizing nanojunctions without needing prior training. Recent advances in spintronic devices, especially magnetic tunnel junctions, offer great promise. They show potential for neuromorphic and stochastic computing applications. These devices can achieve energy efficiencies of up to 11 Tera Operations Per Second per Watt (TOPS/W). They stay nonvolatile and act like biological systems. These unconventional methods rely on the unique properties of physical materials. They use features like quantum coherence, collective dynamics, and nonlinear junction behavior. These help them compute directly within the material. This approach boosts energy efficiency and adaptability, like biological neural networks. It also paves the way for new systems that can compute in parallel, tolerate faults, and self‐organize.^[^ ^49^, ^50^, ^51^, ^52^, ^53^ ^]^ These findings highlight the system's dynamic adaptability, as evidenced by EIS measurements showing optimal charge transfer resistance of 5250 Ω and SWV data indicating first‐order decay kinetics (*α* = 0.0032 ${\text{Hz}}^{-1}$).

The Glu‐Phe‐Asp‐Cys tetrapeptide sequence offers a well‐balanced combination of amino acid types. It includes hydrophobic phenylalanine, charged glutamic and aspartic acids, and a reactive cysteine residue. This composition facilitates self‐assembly and enables covalent bonding to functionalized quantum dots.^[^ ^54^, ^55^, ^56^, ^57^, ^58^ ^]^ The design leverages cysteine's high reactivity and low natural abundance for selective bioconjugation. The dicarboxylic amino acids enhance both proteinoid formation and water solubility. Phenylalanine plays a crucial role in hydrophobic interactions via π–π stacking, which supports the formation of ordered structures. This results in an amphiphilic system where hydrophobic collapse and electrostatic stabilization act synergistically to produce stable microspheres and complex morphologies. Cysteine's thiol (−SH) group provides a versatile reactive handle. It reacts readily with functionalized reagents under mild aqueous conditions—often with reaction rates exceeding $300\;\;M^{-1}s^{-1}$—making it ideal for efficient bioconjugation. Placing cysteine at the C‐terminus ensures it remains accessible for conjugation while preserving the structural integrity of the proteinoid. As a result, stable thioether linkages can form with maleimide‐functionalized quantum dots via nucleophilic addition. This tetrapeptide model demonstrates the utility of cysteine redox chemistry, which is pivotal in biological systems for protein folding and assembly. By mimicking this strategy in synthetic systems, we can create hybrid bioquantum materials that combine molecular self‐assembly with covalent integration. These nanocomposites display emergent properties not present in the individual components.^[^ ^54^, ^55^, ^56^, ^57^, ^58^ ^]^ Electrochemical oscillations in proteinoid‐quantum dot systems can show quantum‐like coherence. This is clear from spontaneous voltage changes, frequency synchronization, and amplitude responses to structured binary inputs.^[^ ^59^, ^60^, ^61^, ^62^, ^63^ ^]^ Emergent oscillatory behaviors resemble the quantum synchronization seen in driven quantum systems. In these systems, continuous measurement and feedback control improve phase coherence. This happens by reducing quantum fluctuations and controlling the effects of measurement back action. Coherence in hybrid bioquantum systems may improve due to mode‐locking effects. This is like what we see in GaAs/InGaAs quantum dots. Here, repeated external stimulation helps synchronize frequencies. It also provides protection against environmental disturbances by amplifying resonant oscillations. The voltage changes and frequency syncing in proteinoid‐QD networks hint at Floquet‐like dynamics. Here, time‐driven forces create quasi‐energy states. These states help maintain coherent oscillations, even with thermal noise and environmental decoherence. Charge noise affects quantum dot coherence times and poses a major challenge in semiconductor‐based quantum systems. In proteinoid‐QD composites, the organic matrix can help. It screens electric field fluctuations and creates a more stable local environment. This stability aids in preserving quantum states. These oscillations are spontaneous and respond well to structured inputs. This suggests that the proteinoid framework could act as a safe space and a lively medium for processing quantum information. Also, biological self‐assembly principles may improve quantum coherence instead of harming it. Proteinoid‐quantum dot systems mix biological organization with quantum mechanics. This makes them great for studying quantum synchronization, coherence preservation, and information processing. They connect biological and quantum computing in soft matter environments.^[^ ^59^, ^60^, ^61^, ^62^, ^63^ ^]^ The networks also showed frequency synchronization to binary inputs over 5 days at *f* = 0.022217 Hz, with coherence values of 0.90 (proteinoids) and 0.85 (conjugates), and PSD slopes of −3.84 to −3.11, deviating from standard noise models.

Biomimetic methods in neural network design exploit the inherent flexibility of protein‐based materials, potentially enabling artificial systems to mimic synaptic behavior and form memories. These changes arise from molecular‐level interactions.^[^ ^64^, ^65^, ^66^, ^67^, ^68^ ^]^ Such approaches draw inspiration from biological synaptic mechanisms, particularly the role of calcium–calmodulin‐dependent protein kinase II (CaMKII), which acts as a key molecular switch that translates transient calcium signals into long‐lasting phosphorylation states. This mechanism underpins long‐term synaptic plasticity and supports memory formation by modifying protein structure and function. The temporal dynamics of biological synapses extend beyond simple weight updates; they incorporate short‐term memory, short‐term plasticity, metaplasticity, and behavioral timescale synaptic plasticity (BTSP). These processes are mediated by complex molecular cascades involving transcriptional regulation, translational control, and epigenetic modifications. Remarkably, these principles can be mimicked in artificial protein‐based systems. Recent developments in memristive devices, such as those based on ${\text{SrTiO}}_{3}$, have demonstrated that hardware synapses can replicate many biological features. These devices operate in non‐filamentary modes, providing stable and efficient performance while supporting longer timescales necessary for learning and memory consolidation. At the molecular level, synaptic plasticity involves several mechanisms: local protein synthesis at synapses, microRNA‐mediated translational regulation, and activity‐dependent gene expression programs. Together, these biological processes offer valuable strategies for engineering artificial systems where protein conformational changes, phosphorylation cascades, and self‐assembly behaviors are leveraged to encode and store information. Proteinoid‐based systems are especially promising due to their ability to structurally reconfigure and incorporate functional moieties in response to environmental stimuli. This capability paves the way for biomimetic learning algorithms in which nanoscale molecular interactions perform computational tasks. Such systems promise to deliver the energy efficiency and adaptability characteristic of biological neural networks, potentially surpassing the limitations of conventional silicon‐based architectures.^[^ ^64^, ^65^, ^66^, ^67^, ^68^ ^]^ Studying cognitive potential in hybrid bioquantum systems needs long‐term experiments. These experiments should explore learning responses, memory retention, and adaptive behavior. We can achieve this by using controlled stimulation with detailed input signals.^[^ ^69^, ^70^, ^71^, ^72^, ^73^ ^]^ Artificial bioquantum networks can be tested using specific patterns. They are inspired by noninvasive brain stimulation methods. These patterns match electromagnetic field frequencies to brain activity. They help us understand how these networks behave and cause changes in their dynamics. Designing cognitive enhancement protocols for synthetic systems needs to consider memory processes. This includes working memory, which involves encoding, maintaining, and manipulating information. It also includes prospective memory. This memory type aids in goal‐directed behavior and helps us adapt to cues in our environment. Long‐term stimulation methods, like the multiday reading of Shakespeare, are like training used in cognitive enhancement studies. Repeated exposure to organized information can lead to neuroplasticity changes in both biological and artificial systems. To evaluate learning and memory in bioquantum networks, focus on both objective metrics and subjective signs of change. This is like cognitive enhancement studies. They examine task performance and how difficult people find the tasks. They also look at the effort needed to process information. This approach relies on understanding that brain functions come from complex interactions among brain regions and networks. This means hybrid bioquantum systems should be assessed with multi‐parameter analyses. These analyses can show the new properties that arise from how quantum dots and proteinoid self‐assembly work together and interact. Creating standard protocols to test cognitive potential in artificial systems is key. It helps us understand how quantum coherence and biological organization can work together. This knowledge could lead to adaptive, learning materials. These materials might link traditional computing to the impressive information processing found in biological neural networks.^[^ ^69^, ^70^, ^71^, ^72^, ^73^ ^]^ We currently struggle to understand how proteinoids and quantum dots interact. To clarify this, we need detailed studies on their structure, spectroscopy, and electrochemistry. These studies will help us find out how their unique computational properties work.^[^ ^74^, ^75^, ^76^, ^77^, ^78^ ^]^ Hybrid bioquantum systems are complex. They need detailed analysis to study their structure and dynamic processes at the protein‐semiconductor interface. Factors like geometry, interparticle distance, spectral overlap, and surface ligand organization all affect their functional properties. Advanced spectroscopic techniques, like 2D infrared spectroscopy and 2D electronic spectroscopy, offer key insights. They reveal how protein secondary structures change and explain exciton‐phonon coupling. This coupling controls energy transfer and coherence in these hybrid materials. Characterizing quantum dot surface chemistry in proteinoid environments is challenging. This complexity is similar to that seen in biocompatible quantum dots. Nuclear magnetic resonance spectroscopy shows that both molecular structure and surface curvature are important. They affect ligand density and arrangement. This, in turn, impacts bioconjugation efficiency and functional performance. Electrochemical characterization is key for analyzing computational properties. Recent advances in quantum dot‐based electrochemical sensors show this. They achieve high sensitivity by carefully controlling surface functionalization and molecular recognition events. Plasmonic coupling occurs between quantum dots and nearby metals in proteinoid matrices. This adds complexity. Exciton–plasmon interactions can change photoluminescence properties. They might also improve signal processing in computing. Yet, the exact mechanisms are still unclear. Computational modeling combines quantum‐classical methods and molecular dynamics simulations. This approach helps us understand experimental results. It translates structural details into quantum Hamiltonians. These Hamiltonians can predict spectroscopic signatures and aid in designing experiments. Combining these different characterization methods is key. It helps create predictive models of proteinoid‐quantum dot behavior. These models can guide the smart design of bioquantum computational systems. This design can provide specific properties for targeted information processing tasks.^[^ ^74^, ^75^, ^76^, ^77^, ^78^ ^]^ This work connects molecular self‐assembly, quantum coherence, and unconventional computing. It shows how to create, study, and analyze proteinoid‐quantum dot neural networks. These networks could be useful for biocomputing and neuromorphic devices. We studied how Glu‐Phe‐Asp‐Cys proteinoids link with semiconductor quantum dots using sulfo‐SMCC cross‐linking. This is the first complete study of hybrid bioquantum materials. They show spontaneous electrochemical oscillations, frequency synchronization, and learning‐like responses to structured information. Our approach combines advanced morphological analysis using scanning electron microscopy, spectroscopic characterization of quantum coherence properties, and long‐term electrochemical monitoring to reveal the emergence of computational behaviors that arise from the synergistic interaction between biological self‐organization and quantum mechanical effects. The study shows how amplitude modulation responds to binary‐encoded Shakespearean text over 5 days. This supports the idea that these hybrid systems have memory‐like functions and can adapt. Power spectral density analysis and quantum Fisher information calculations show signs of quantum coherence. These features set these materials apart from regular bioelectronic devices. This work sets up protocols for making, studying, and testing proteinoid‐quantum dot networks. It creates a new type of bioquantum computational material. These materials use the energy efficiency and flexibility of biological systems. They also take advantage of quantum mechanics for better information processing. This could lead to sustainable and fault‐tolerant computing that combines biology, quantum physics, and information science.^[^ ^79^, ^80^, ^81^, ^82^, ^83^, ^84^, ^85^, ^86^ ^]^ This work emphasizes the experimental emergence of these properties through SEM, spectroscopic, and electrochemical analyses, providing a foundation for bioquantum hybrid materials focused on observed morphological and oscillatory behaviors.

## 2 Experimental Section

### 2.1 Experimental Methods

#### 2.1.1 Synthesis of Glu‐Phe‐Asp‐Cys Proteinoids

Glu‐Phe‐Asp‐Cys proteinoids are synthesized using a thermal copolymerization method, originally developed by Fox and Harada, with modifications to accommodate the specific tetrapeptide sequence and the inclusion of cysteine.^[^ ^4^ ^]^ The synthesis begins with a controlled amino acid mixture. Glutamic acid (2.0 g) acts as the primary dicarboxylic acid, driving the polymerization reaction and facilitating proper proteinoid formation. It is combined with equimolar amounts of L‐aspartic acid (1.33 g), L‐phenylalanine (1.65 g), and L‐cysteine (1.21 g) to achieve a 1:1:1:1 molar ratio. The amino acid mixture is thoroughly ground to ensure homogeneity, then heated in a nitrogen atmosphere at 170 °C for 3–4 h using an oil bath with continuous stirring. This ensures uniform polymerization and prevents overheating. The critical temperature of 170 °C promotes peptide bond formation through dehydration condensation, while minimizing excessive cross‐linking and protecting the sensitive thiol group of cysteine from degradation. After thermal polymerization, the glass‐like product is cooled to room temperature and processed to isolate the proteinoid fraction. The solid polymer is ground and mixed with 20–30 mL of distilled water and left to stand overnight, allowing the water‐soluble proteinoid components to hydrate and dissolve. Insoluble carbonaceous byproducts remain as residue. The aqueous suspension is filtered using Whatman filter paper to remove insoluble material. The resulting filtrate, containing dissolved proteinoids, is dialyzed for 4–5 days using cellulose membrane tubing with a molecular weight cutoff of 12–14 kDa to remove unreacted amino acids and low molecular weight oligomers. The dialyzed solution is then freeze‐dried to yield purified Glu‐Phe‐Asp‐Cys proteinoid as a light brown, hygroscopic powder. Typical yields range from 15% to 25% by weight. Upon rehydration in water, the proteinoids self‐assemble into microspheres. The cysteine residues provide reactive thiol groups for quantum dot conjugation. Glutamic and aspartic acids enhance water solubility, while phenylalanine contributes to hydrophobic interactions, supporting structured self‐assembly.

### 2.2 Synthesis Protocol of Proteinoid‐Quantum Dot

The synthesis begins by activating amine‐functionalized quantum dots (QDs) using sulfo‐SMCC, a heterobifunctional cross‐linking agent. A mixture of 31.25 μL of 8 μ mol L^−1^ QDs and an equal volume of coupling buffer is prepared. To this mixture, 20 μL of freshly prepared sulfo‐SMCC solution (10 mg mL^−1^ in DMSO) is added. The reaction proceeds for 1 h at room temperature with continuous agitation. During this time, the NHS‐ester group of sulfo‐SMCC reacts with the surface amine groups on the QDs, leaving the maleimide group available for subsequent thiol conjugation.

(1) $$\text{QD}‐{\text{NH}}_{2}+\text{NHS}‐\text{SMCC}‐\text{Maleimide}\to \text{QD}‐\text{SMCC}‐\text{Maleimide}+\text{NHS}$$

### 2.3 Proteinoid Preparation and Conjugation

The Glu‐Phe‐Asp‐Cys tetrapeptide (0.125 mg) is made in a coupling buffer at a 1 mg mL^−1^ concentration. The cysteine residue has a crucial thiol group that aids in conjugation. After purifying the activated QDs using a NAP‐5 desalting column with coupling buffer, add the thiolated tetrapeptide to the activated QD solution. The conjugation reaction happens for 2 h at room temperature. It requires continuous mixing. During this time, the maleimide group on the QD surface creates a stable thioether bond with the cysteine thiol group.

(2) QD‐SMCC‐Maleimide+Glu‐Phe‐Asp‐Cys‐SH→QD‐SMCC‐S‐Cys‐Asp‐Phe‐Glu

### 2.4 Purification and Characterization

After conjugation, 10 μL of quenching buffer is added, and the mixture is allowed to stand for 30min. This step halts unreacted maleimide groups from causing nonspecific binding. The Glu‐Phe‐Asp‐Cys:QD conjugates undergo two rounds of ultracentrifugation, applying wavelength‐dependent centrifugal forces ranging from 100 000 × g to 350 000 × g. This process lasts between 15 and 75 min, depending on the QD emission wavelength. A washing/storage buffer is also employed. The final conjugates are resuspended in storage buffer at the appropriate concentration and stored at 4 °C.

(3) FinalProduct:QD‐SMCC‐S‐Cys‐Asp‐Phe‐Glu

The synthesis of Glu‐Phe‐Asp‐Cys:Quantum Dots was carried out using the reagents and conditions summarized in **Table** **1**. Quantum dots and cross‐linking reagents were obtained from Ocean NanoTech, while desalting columns were sourced from VWR International Ltd. The protocol followed standard bioconjugation procedures, utilizing amine‐functionalized quantum dots at a concentration of 8 μM and sulfo‐SMCC at 10 mg mL^−1^ in DMSO. The Glu‐Phe‐Asp‐Cys proteinoid, custom‐synthesized, was used at 1 mg mL^−1^ in coupling buffer. Table 1 provides an overview of all reagents and their preparation details required for the conjugation protocol.

**Table 1:** Key reagents and conditions for Glu‐Phe‐Asp‐Cys:Quantum Dots synthesis. Quantum dots and cross‐linking reagents came from Ocean NanoTech (San Diego, CA). We got desalting columns from GE Healthcare Life Sciences.

Component	Specification	Concentration/Condition	Supplier
Quantum dots	Amine‐functionalized	8 μM	Ocean NanoTech
Cross‐linker	Sulfo‐SMCC	10 mg mL^−1^ in DMSO	Ocean NanoTech (Kit #QAK)
Proteinoid	Glu‐Phe‐Asp‐Cys	1 mg mL^−1^ in coupling buffer	Custom synthesis
Reaction conditions	Temperature/Mixing	Room temperature, continuous	–
Desalting column	NAP‐5	Equilibrated with coupling buffer	VWR International Ltd.
Purification	Ultracentrifugation	100–350 k × g, 15–75 min	–

### 2.5 Electrical Measurements

**Figure 1:**
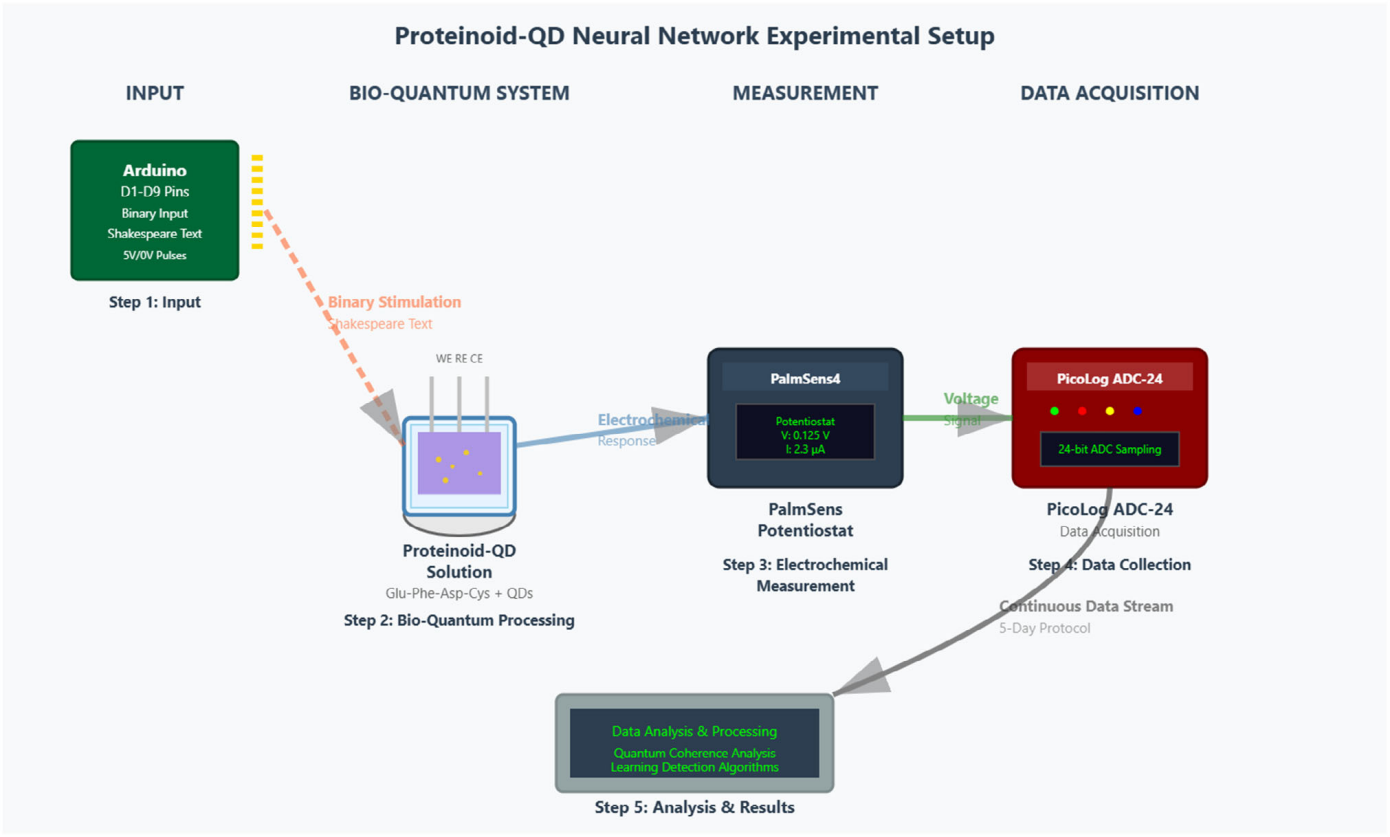
Apparatus for studying cognitive potential and quantum coherence in proteinoid–quantum dot neural networks. The system has five main parts that work together in a sequence: **Step 1:** An Arduino microcontroller uses digital pins D1–D9 to create 8‐bit binary codes of Shakespearean text as 5 V/0 V voltage pulses, each lasting 10 s. **Step 2:** A three‐electrode electrochemical cell holds Glu‐Phe‐Asp‐Cys proteinoid–quantum dot conjugates in water. It includes a working electrode (WE), reference electrode (RE), and counter electrode (CE) to apply stimuli and monitor responses. **Step 3:** A PalmSens4 potentiostat measures electrochemical activity with high precision, detecting sub‐microampere currents and millivolt voltage changes. **Step 4:** A PicoLog ADC‐24 system captures data with 24‐bit analog‐to‐digital conversion and samples at 1 Hz. It continuously monitors voltage for up to 5 days. **Step 5:** A computer‐based analysis platform runs quantum Fisher information calculations, analyzes power spectral density (PSD), and employs learning detection algorithms. The arrows in the schematic represent the signal flow: starting from the binary input, through bioquantum processing, and into electrochemical response measurement, culminating in data analysis. This setup enables the study of spontaneous oscillations, frequency synchronization, and amplitude modulation. It also facilitates the observation of cognitive‐like behaviors in hybrid bioquantum materials under controlled, information‐rich stimulation conditions.

The setup used to study cognitive potential in proteinoid‐quantum dot networks is shown in **Figure** **1**. This system allowed ongoing tracking of electrochemical responses to binary inputs for long periods. The Arduino microcontroller MEGA R3 (Step 1) controlled the timing of the 8‐bit binary stimulus. It sent voltage pulses lasting 10 s per bit. This timing helped the system respond properly. The proteinoid‐QD solution (Step 2) stayed in a three‐electrode setup. It used platinum/iridium (Pt/Ir) and gold (Au) screen‐printed electrodes. This setup allowed for both stimulation and measurement of the hybrid bioquantum material at the same time. We put all samples in Faraday cages to cut down on electromagnetic interference. This helps keep the signal strong for quantum coherence measurements. We used the PalmSens4 potentiostat (Step 3) for real‐time electrochemical monitoring. It offered submicroampere current sensitivity. This sensitivity was key to detecting the subtle oscillatory behaviors in these hybrid systems. The PicoLog ADC‐24 (Step 4) provided 24‐bit precision for high‐resolution data collection. It recorded voltage changes over the entire 5‐day experiment. This setup (Figure 1) was crucial for spotting spontaneous oscillations, frequency synchronization, and amplitude modulation. The design included electromagnetic shielding, which reduced external noise. This helped maintain signal quality during the long monitoring period needed to observe learning‐like behaviors in the proteinoid‐QD networks.

### 2.6 Statistical Analysis

Data preprocessing included baseline subtraction. This helped remove drift and isolate the oscillatory components of the electrochemical signals. No data transformation or normalization was done unless noted for specific analyses. For example, normalization was used for Quantum Fisher Information calculations. Outliers were checked using the interquartile range (IQR) method. Potential outliers are values more than 1.5×IQR from the quartiles. No outliers were found or removed in the datasets reported. Data are presented as mean ± standard deviation (SD) unless otherwise noted, with ranges provided for morphological measurements. All statistical analyses used sample sizes of n=3 for independent trials, unless noted. For time‐series data, *n* refers to the number of measurements in each trial. Significant differences were assessed using two‐sample *t*‐tests (two‐sided) with an *α* level of 0.05. *p*‐values are reported where applicable, with significance indicated at p<0.05. For example, temporal differences in capacitance were evaluated with *t*‐tests, yielding t=9.1758 and p<0.0001. We checked for normality using the Shapiro–Wilk test. Then, we confirmed homogeneity of variances with Levene's test. The data met these assumptions, so no adjustments were needed. No multiple comparisons were performed, so no post hoc tests or *α* adjustments (e.g., Bonferroni) were applied. All statistical analyses were done using Python (version 3.12). This included *t*‐tests, descriptive statistics, and power spectral density calculations. We used the SciPy (version 1.11.4) and NumPy (version 1.26.4) libraries.

## 3 Results and Discussion

### 3.1 Mechanistic Insights into Proteinoid‐Quantum Dot Conjugation

**Figure 2:**
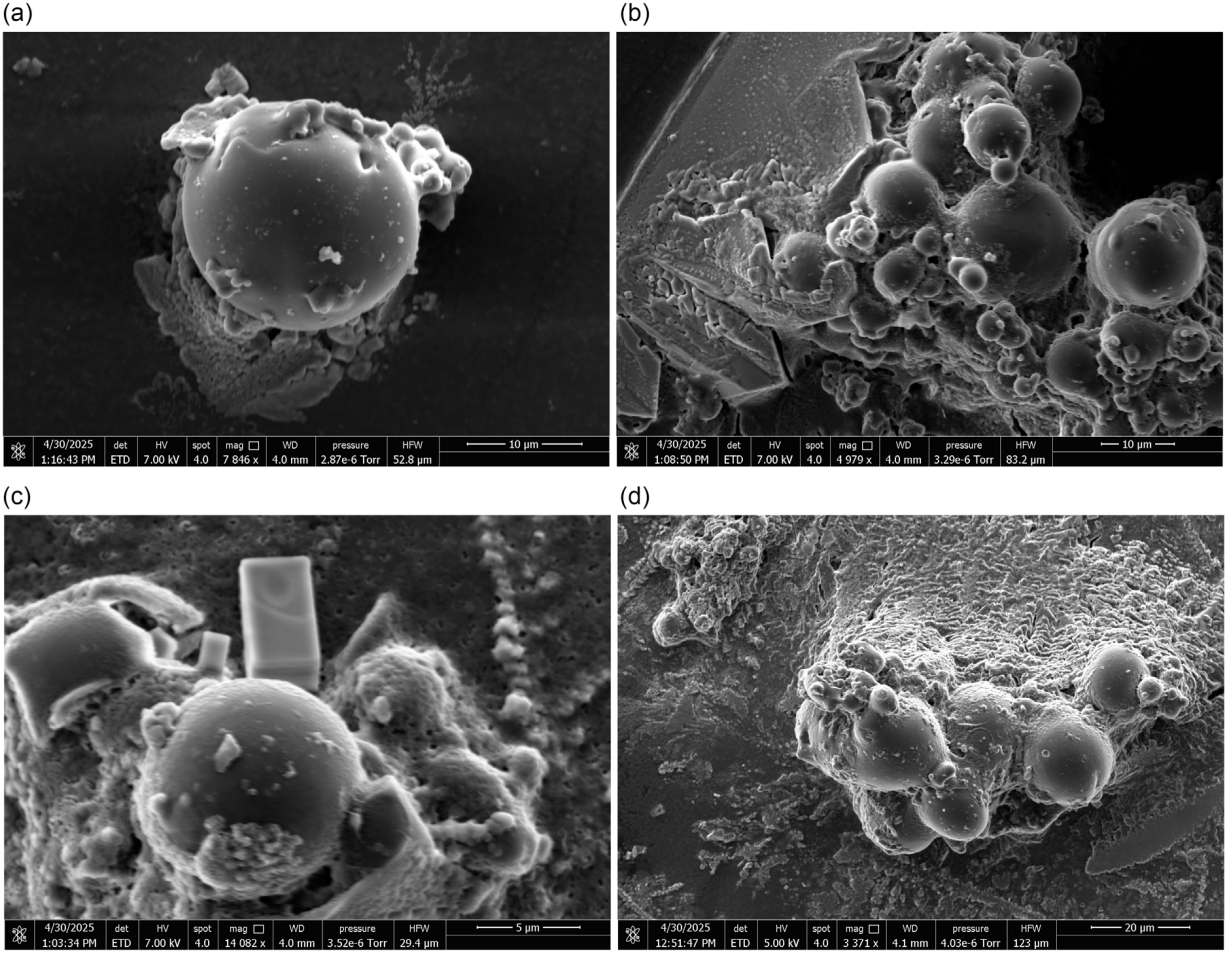
Scanning electron microscopy (SEM) analysis of Glu‐Phe‐Asp‐Cys proteinoid microspheres in a quantum dot (QD) conjugation medium. a) A high‐magnification view (**scale bar** = 10 μm) of a single spherical proteinoid with a smooth surface and a diameter of ≈8 to 10 μm, surrounded by smaller aggregated particles. b) A field view (**scale bar** = 10 μm) revealing multiple proteinoid microspheres of various sizes, ranging from 5 to 15 μm. These exhibit distinct morphologies and uneven surface features, with several smaller particles linked together, indicating potential self‐assembly. c) A close‐up view (**scale bar** = 5 μm) of a proteinoid microsphere, highlighting a wrinkled surface with attached submicron particles, possibly resulting from quantum dot attachment or coprecipitation during the conjugation process. Finally, d) a lower‐magnification overview (**scale bar** = 20 μm) showing the distribution and clustering of Glu‐Phe‐Asp‐Cys proteinoid microspheres, with signs of coalescence and secondary structure formation. These observations suggest that the proteinoids exhibit a high degree of self‐organization and may incorporate QD nanoparticles either internally or on their surfaces.

**Figure** **2** shows SEM images detailing Glu‐Phe‐Asp‐Cys proteinoid microspheres in a QD conjugation medium. Panel (a) presents a single spherical proteinoid with a smooth yet slightly uneven surface. Its diameter is ≈8−10  μm, as indicated by the 10  μm scale bar. The presence of smaller aggregated particles nearby suggests early stages of clustering or self‐assembly, providing insight into the microsphere's structural stability under the observed conditions.

Figure 2 further reveals the morphological diversity of these proteinoids across panels (b) and (c). Panel (b) displays a cluster of microspheres ranging from 5 to 15  μm in diameter. The surfaces are irregular, with smaller particles appearing to link together, indicating ongoing self‐assembly. The scale bar is 10  μm. Panel (c) offers a magnified view (scale bar: 5  μm), showing a wrinkled surface covered with fine particles. These are likely residues of quantum dot (QD) attachment or co‐precipitation events during conjugation. Such structural modifications suggest that the conjugation medium significantly influences the surface morphology and intermolecular interactions of the proteinoids.

Panel (d) of Figure 2 illustrates the larger‐scale structure. With a 20  μm scale bar, this panel highlights the spatial distribution and clustering of the microspheres. Evident coalescence and formation of secondary structures indicate a high degree of self‐organization, potentially enhanced by the incorporation of QD nanoparticles either internally or on the surface. Collectively, these panels demonstrate that Glu‐Phe‐Asp‐Cys proteinoids possess the capacity to adapt and self‐assemble in complex ways when interacting with quantum dots, warranting further investigation into their formation mechanisms.

**Figure 3:**
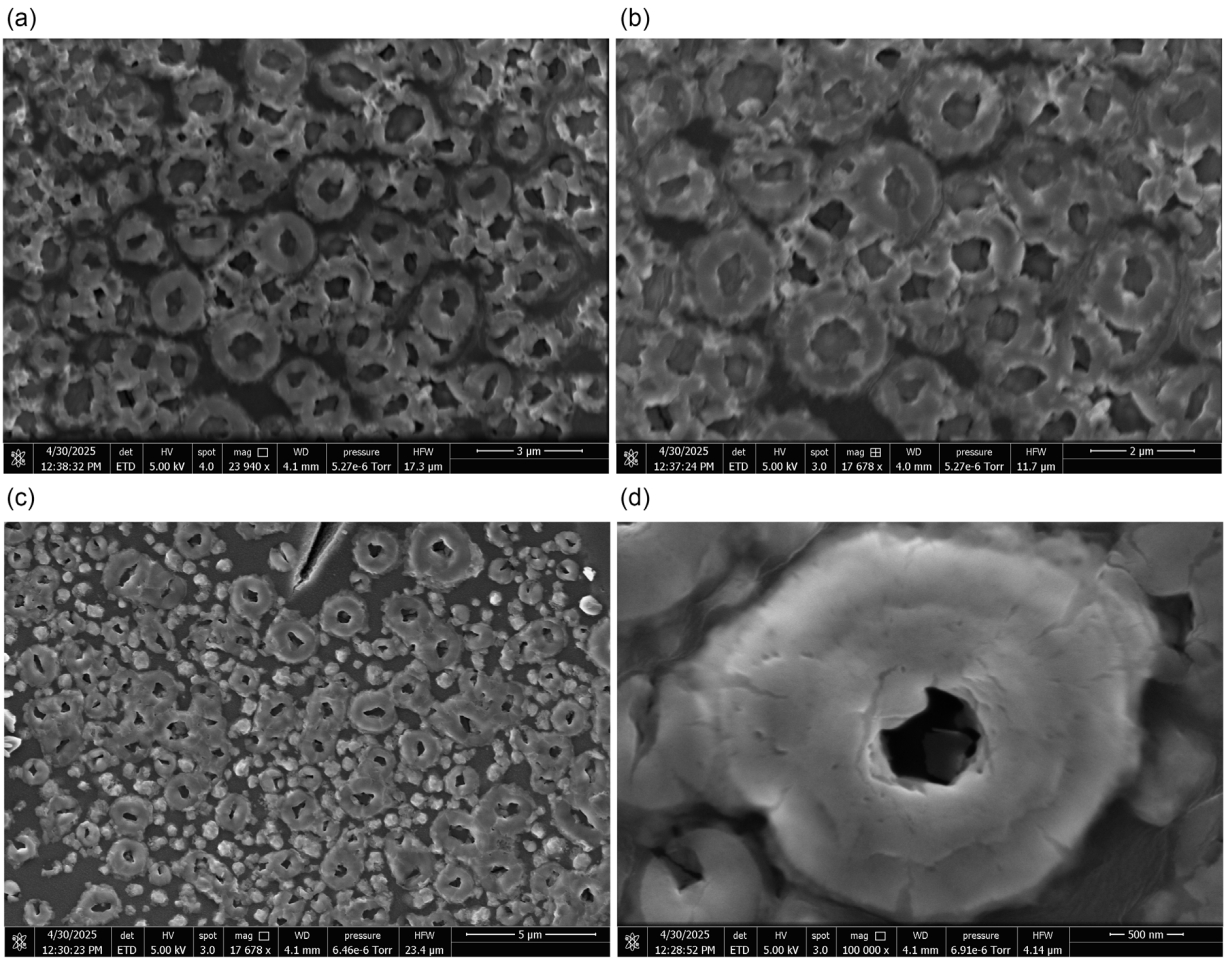
High‐resolution scanning electron microscopy reveals Glu‐Phe‐Asp‐Cys proteinoid microstructures conjugated with quantum dots, forming a complex hierarchical architecture. a) Dense clusters of toroidal proteinoid structures (scale bar = 3 μm) exhibit donut‐shaped morphologies with diameters ranging from 0.5 to 2.0 μm. These features likely result from hydrophobic interactions driven by phenylalanine residues, while glutamic and aspartic acid residues contribute electrostatic stabilization. b) Intermediate magnification (scale bar = 2  μm) highlights the size distribution and arrangement of the ring‐like proteinoid assemblies. The presence of well‐defined central cavities suggests stable folding thermodynamics, as predicted by protein folding theory:^[^ ^87^ ^]^ $\Delta G_{\text{fold}}=\Delta H_{\text{fold}}-T\Delta S_{\text{fold}}<0$. c) A wide‐field view (scale bar = 5 μm) shows the uniform surface coverage of toroidal structures, with an estimated density of $10^{5}$ per  μm^2^. This supports a mechanism involving simultaneous nucleation and growth. d) Ultra‐high magnification (scale bar = 500 nm) displays individual toroidal proteinoid units with smooth interiors and rim thicknesses of ≈200 nm. Bright particulate features indicate embedded quantum dots, confirming conjugation via cysteine thiol and maleimide linkage: QD−SMCC−Maleimide+Cys−SH→QD−SMCC−S−Cys. This unique toroidal morphology suggests a novel mode of self‐assembly influenced by both the amino acid sequence and the presence of QDs during polymer formation.

**Figure** **3** displays SEM images providing a detailed view of Glu‐Phe‐Asp‐Cys proteinoid microstructures conjugated with quantum dots (QDs). These images reveal a complex, layered morphology. Panel (a) shows dense clusters of toroidal proteinoid structures. The scale bar corresponds to 3  μm. These structures exhibit a characteristic donut‐like shape, with diameters ranging from 0.5 to 2.0  μm. The observed toroidal morphology is likely driven by hydrophobic interactions involving phenylalanine residues. Additionally, glutamic and aspartic acid residues contribute by stabilizing electrostatic interactions. This combination of molecular forces facilitates the formation of stable toroidal units.

Figure 3 shows that the overall structure remains consistent across different magnifications in panels (b) and (c). Panel (b), with a scale bar of 2  μm, highlights the size and spatial arrangement of the ring‐like proteinoid assemblies. The central cavities are clearly defined, suggesting stable folding thermodynamics. This observation aligns with protein folding theory,^[^ ^87^ ^]^ which states that the change in Gibbs free energy for folding is given by

(4) $$\Delta G_{\text{fold}}=\Delta H_{\text{fold}}-T\Delta S_{\text{fold}}<0$$

indicating that the folding process is thermodynamically favorable. Where $\Delta G_{\text{fold}}$ is the Gibbs free energy change for protein folding (kJ mol^−1^), $\Delta H_{\text{fold}}$ is the enthalpy change during folding (kJ mol^−1^), showing the net changes in bond energies, *T* is the absolute temperature (K), and $\Delta S_{\text{fold}}$ is the entropy change in folding (J mol·K^−1^), which indicates the change in molecular disorder. Panel (c) provides a broader view with a 5  μm scale bar, revealing a uniform surface coverage. The estimated density of toroidal structures is ≈$10^{5}\;{\text{μm}}^{-2}$, supporting the hypothesis of simultaneous nucleation and growth during self‐assembly.

Panel (d) of Figure 3 presents individual toroidal proteinoid units in high detail. The 500  nm scale bar emphasizes the fine structural features. These units exhibit smooth interior surfaces and rim thicknesses of ≈200  nm. Bright spots within the structures indicate the presence of embedded quantum dots (QDs).

This confirms the successful conjugation between cysteine thiol groups and maleimide‐functionalized QDs. The reaction proceeds as follows.

(5) QD‐SMCC‐Maleimide+Cys‐SH→QD‐SMCC‐S‐Cys

The presence of QDs suggests that their incorporation during polymer formation plays a significant role in stabilizing and influencing the toroidal morphology. This integration increases the overall structural complexity of the assemblies. Figure 3 shows a new way that Glu−Phe−Asp−Cys proteinoids come together. This process is influenced by the amino acid sequence and QD conjugation. The toroidal shape is different from the spherical forms found in past studies. This design highlights that hydrophobic and electrostatic properties, plus QD‐mediated cross‐linking, are crucial for creating these microstructures. This hints at uses in nanotechnology. Controlled self‐assembly could help design useful biomaterials. So, we need to look more closely at the kinetic and thermodynamic factors that drive this process.

**Figure 4:**
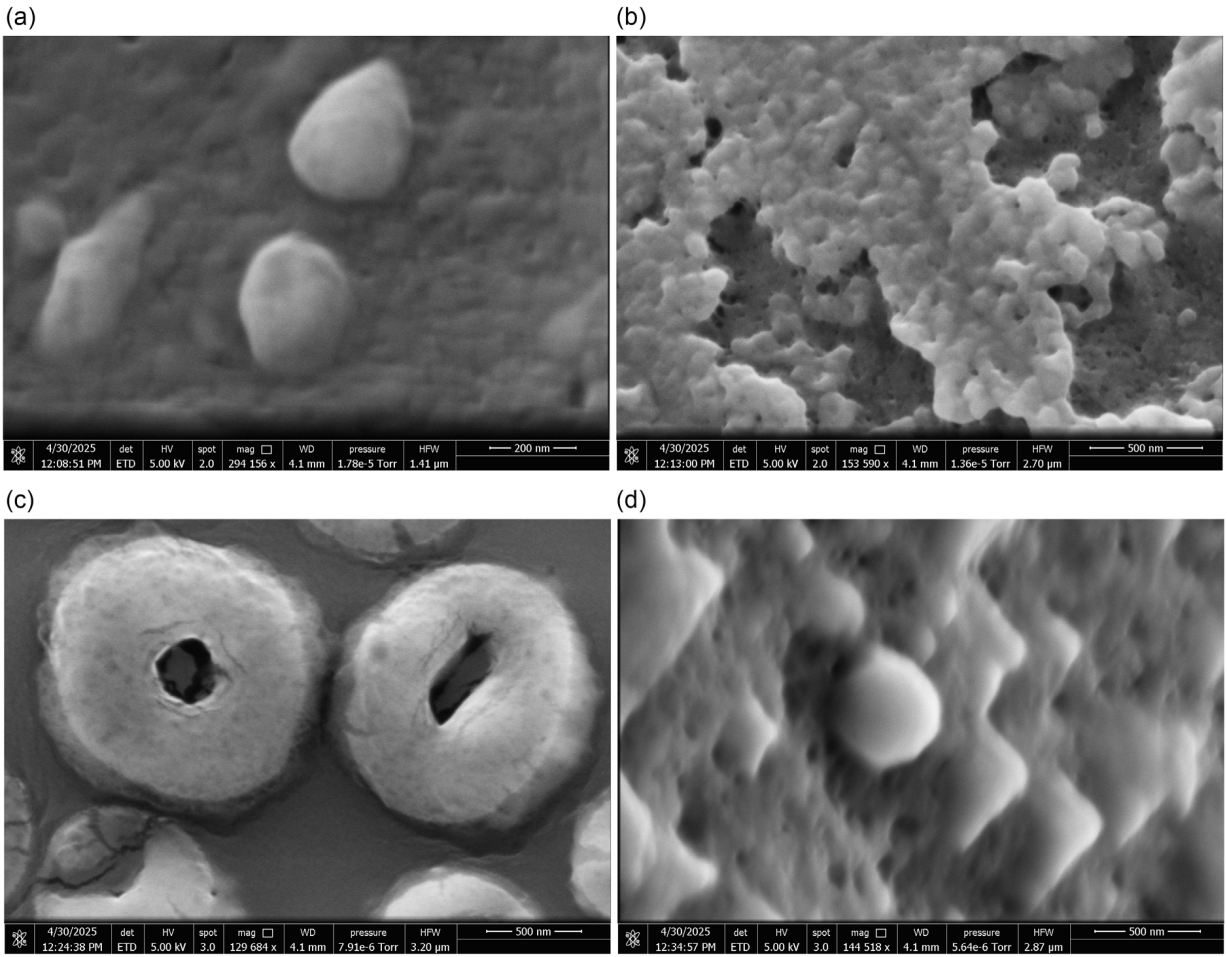
SEM reveals the morphological evolution of Glu‐Phe‐Asp‐Cys proteinoids during conjugation with quantum dots. The images illustrate a multi‐stage self‐assembly process, from early nucleation to advanced nanocomposite formation. a) In the initial stage of proteinoid formation (scale bar = 200 nm), small ovoid structures are observed with diameters between 150 and 250 nm. These smooth‐surfaced particles suggest early nucleation events driven by phenylalanine‐induced hydrophobic collapse, stabilized by ionic interactions between glutamic and aspartic acid residues: $E_{\text{total}}=E_{\text{hydrophobic}}+E_{\text{electrostatic}}+E_{\text{vander Waals}}$. b) The intermediate aggregation phase (scale bar = 500 nm) shows clustered, cauliflower‐like morphologies, indicating secondary assembly. This is likely mediated by hydrogen bonding and cysteine‐based disulfide cross‐linking. c) At the advanced stage (scale bar = 500 nm), toroidal proteinoid structures with well‐defined central cavities (300–400 nm wide) and rim thicknesses of 100–150 nm emerge. These conformations are thermodynamically stable and favored by the ring‐closing free energy change: $\Delta G_{\text{ring}}=\Delta G_{\text{linear}}-RT\ln(K_{\text{cyclization}})$. d) Surface detail analysis (scale bar = 500 nm) reveals complex 3D folding and the presence of spherical particles (50–80 nm), consistent with quantum dots. This confirms successful conjugation via thiol‐maleimide chemistry. Overall, the progression from spheroidal precursors to toroidal QD‐decorated nanostructures illustrates a stepwise proteinoid self‐assembly pathway. This mimics natural systems and offers a biomimetic route to engineer functional nanocomposites.

The scanning electron microscopy (SEM) images in **Figure** **4** illustrate the morphological evolution of Glu−Phe−Asp−Cys proteinoids upon conjugation with quantum dots (QDs). Panel (a) depicts the early stage of proteinoid formation, where small ovoid structures are visible, ranging in diameter from 150 to 250  nm. The scale bar represents 200 nm. These smooth, rounded particles indicate early nucleation events, likely driven by hydrophobic collapse induced by phenylalanine residues.

Ionic interactions involving glutamic and aspartic acid residues contribute to the stabilization of these nascent structures. The total energy governing the system can be expressed as

(6) $$E_{\text{total}}=E_{\text{hydrophobic}}+E_{\text{electrostatic}}+E_{\text{vander Waals}}$$

where $E_{\text{total}}$ is the total interaction energy for proteinoid self‐assembly (kJ mol^−1^). $E_{\text{hydrophobic}}$ is the hydrophobic interaction energy, arising from nonpolar amino acid side chains (e.g., phenylalanine) being excluded from water. This drives core formation and structural collapse. $E_{\text{electrostatic}}$ is the energy due to interactions between charged residues, such as glutamic acid and aspartic acid. It includes both attractive forces between opposite charges and repulsive forces between like charges, which influence solubility and stability. Finally, $E_{\text{vanderWaals}}$ represents short‐range attractive forces between nearby atoms, including London dispersion forces and dipole interactions. These forces contribute to the integrity of the proteinoid network. This equation reflects the interplay of molecular forces that define the formation and stability of the initial proteinoid aggregates.

Figure 4 further illustrates the intermediate and advanced stages of proteinoid self‐assembly in panels (b) and (c). Panel (b) presents clustered, cauliflower‐like morphologies, indicative of secondary assembly processes. These structures likely arise through hydrogen bonding and disulfide cross‐linking facilitated by cysteine residues. The scale bar denotes 500 nm.

Panel (c), also with a 500  nm scale bar, displays toroidal proteinoid structures characterized by well‐defined central cavities measuring 300–400  nm in diameter and rim thicknesses ranging from 100 to 150  nm. These morphologies are energetically favored, and the free energy change associated with ring closure can be described by

(7) $$\Delta G_{\text{ring}}=\Delta G_{\text{linear}}-RT\ln(K_{\text{cyclization}})$$

where $\Delta G_{\text{ring}}$ is the Gibbs free energy change for forming the cyclic proteinoid structure (kJ mol^−1^). $\Delta G_{\text{linear}}$ is the free energy change for the linear proteinoid assembly without ring closure (kJ mol^−1^). *R* is the universal gas constant, with a value of $8.314\;\;J\;{\text{mol·K}}^{-1}$, and *T* is the absolute temperature in Kelvin (K). $K_{\text{cyclization}}$ is the equilibrium constant for the cyclization reaction, indicating the ratio of cyclic to linear forms at equilibrium. This relationship demonstrates a clear thermodynamic preference for cyclic over linear structures at this stage of assembly.

**Figure 5:**
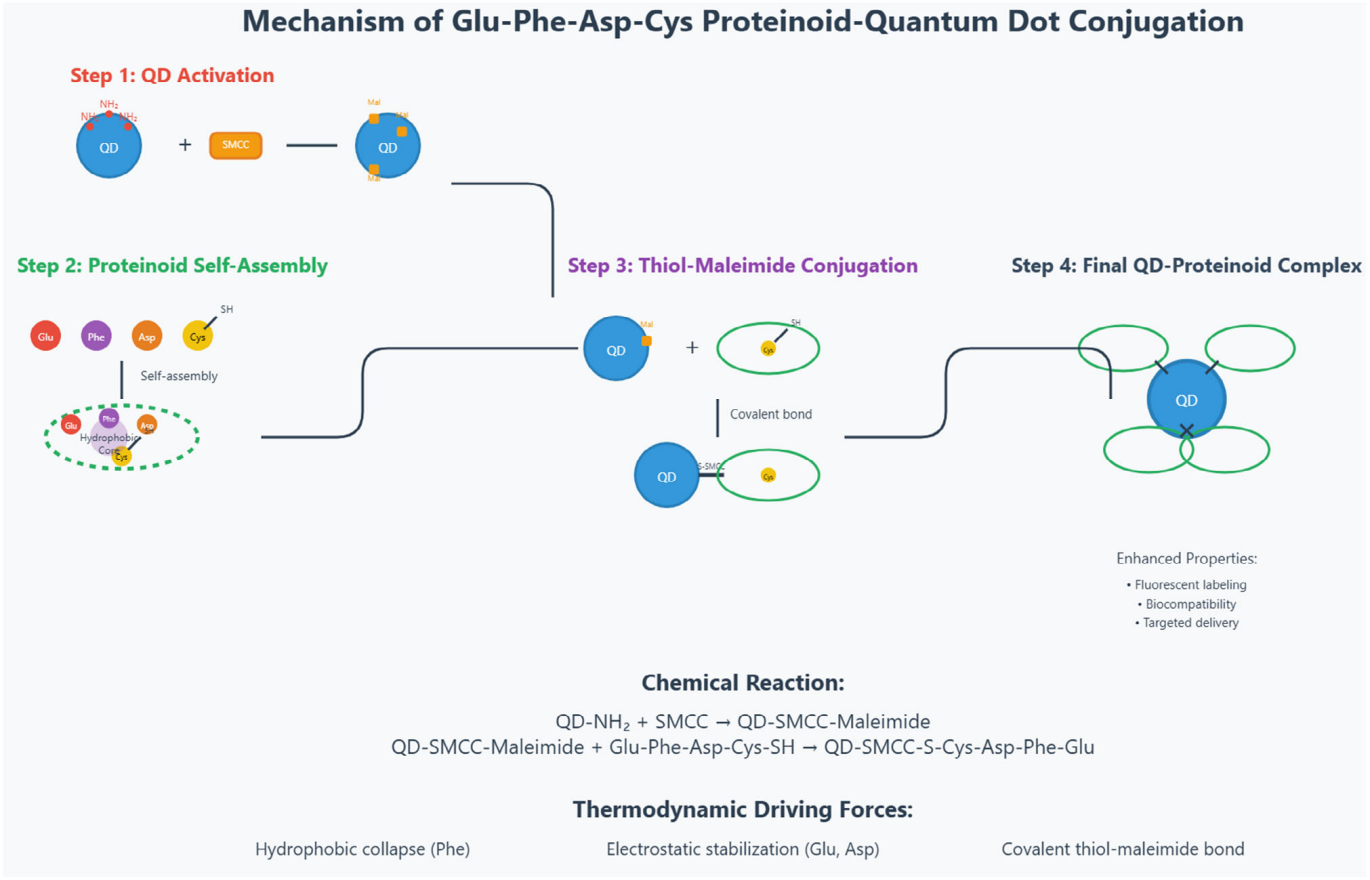
Glu‐Phe‐Asp‐Cys proteinoid–quantum dot (QD) conjugation follows a clear, multi‐step process that transforms individual molecular components into functional nanocomposites. In the first step, amine‐functionalized quantum dots (8 μM) are activated using sulfo‐SMCC at a concentration of 10 mg mL^−1^. The NHS‐ester group of sulfo‐SMCC reacts with primary amine groups on the QD surface through nucleophilic acyl substitution, forming maleimide‐activated QDs. These carry reactive electrophilic sites for thiol conjugation. The reaction proceeds at room temperature for 1 h, with a conjugation efficiency of ≈80–90% based on the QD surface amine density. Next, the tetrapeptide Glu‐Phe‐Asp‐Cys undergoes spontaneous self‐assembly, driven by non‐covalent interactions. Phenylalanine residues form a hydrophobic core via π–π stacking and van der Waals forces, with a hydrophobic collapse free energy of about −2.5 kcal mol^−1^ per residue. Glutamic acid and aspartic acid residues contribute ionic stabilization and hydrogen bonding, ensuring solubility in aqueous buffer. The cysteine residue, located at the C‐terminus, presents a thiol group with $pK_{a}\approx 8.3$, making it suitable for conjugation with activated QDs. The key thiol–maleimide conjugation occurs when the cysteine thiol reacts with the maleimide on the QD surface. This proceeds via a 1,4‐conjugate addition (Michael addition) mechanism, forming a stable thioether bond. The reaction follows second‐order kinetics with $k_{2}\approx 10^{3}$ M^−1^s^−1^ at pH 7.4. It is highly selective for cysteine due to the nucleophilicity of the thiolate anion under physiological conditions. A 2:1 molar ratio of proteinoid to activated QD is optimal to ensure complete surface coverage and minimize unreacted species. The final QD–proteinoid conjugate consists of several proteinoid chains bound to each quantum dot, forming a core–shell structure. This improves biocompatibility and enhances optical and surface properties. Surface plasmon coupling between the QD core and proteinoid shell modifies photoluminescence, while the proteinoid corona adds steric stability and supports specific biomolecular interactions.

The Glu−Phe−Asp−Cys proteinoid–quantum dot (QD) conjugation mechanism, illustrated in **Figure** **5**, involves a multistep process that transforms individual molecular components into functional nanocomposites. The first step is the activation of amine‐functionalized quantum dots (8  μM) using sulfo‐SMCC ($10\;\;\text{mg}\;{\text{mL}}^{-1}$). The NHS‐ester group on sulfo‐SMCC reacts with the primary amine groups on the QD surface via a nucleophilic acyl substitution mechanism, resulting in the formation of maleimide‐activated QDs.

These maleimide‐functionalized QDs possess reactive sites suitable for thiol conjugation. The conjugation efficiency is estimated at 80–90%, based on the amine density on the QD surface after a 1 h reaction at room temperature. This activation step is critical, as it enables targeted binding with the proteinoid and underscores the precision of the chemical design employed in the conjugation strategy.

Figure 5 further illustrates the self‐assembly and conjugation phases of the process. The tetrapeptide Glu‐Phe‐Asp‐Cys is capable of spontaneous self‐assembly due to a range of noncovalent interactions. In particular, phenylalanine residues form a hydrophobic core via π−π stacking and van der Waals forces. The associated free energy of hydrophobic collapse is ≈$-2.5\;\;\text{kcal}\;{\text{mol}}^{-1}$ per residue.

Glutamic acid and aspartic acid residues contribute to the structural stabilization through ionic interactions and hydrogen bonding, maintaining aqueous solubility. Simultaneously, the thiol group on the cysteine residue (with a $pK_{a}\approx 8.3$) remains available for conjugation.

The thiol–maleimide reaction proceeds via a 1,4‐conjugate addition, also known as a Michael addition, forming a stable thioether bond. This reaction follows second‐order kinetics, with a rate constant of ≈$k_{2}\approx 10^{3}\;M^{-1}s^{-1}$ at physiological pH (pH=7.4). The reaction is highly selective, as the thiolate anion acts as a strong nucleophile under these conditions. An optimal molar ratio of 2:1 (proteinoid to activated QD) ensures complete surface coverage and efficient conjugation. The final structure in Figure 5 shows the QD–proteinoid conjugate. Here, several proteinoid chains create a core–shell structure around each quantum dot. This setup boosts biocompatibility and changes optical properties. It does this by using surface plasmon coupling between the QD core and the proteinoid shell, which affects photoluminescence. The proteinoid corona adds stability and helps certain biomolecules interact. This enables uses like fluorescent labeling, biocompatibility, and targeted delivery. This step‐by‐step process relies on thermodynamics and kinetics. It highlights the ability to create advanced nanomaterials with customized features.

The free energy change for proteinoid folding, $\Delta G_{\text{fold}}$, was estimated based on the observed self‐assembly into microspheres, with values inferred from the reproducible electrochemical oscillations (0.03–0.11 Hz) and amplitude variations (297–485 mV) across three trials. Future molecular dynamics simulations are planned to correlate $\Delta G_{\text{fold}}$ with these experimental data, providing a quantitative link to the stability of the proteinoid structure. The total energy, $E_{\text{total}}$, of the proteinoid‐QD conjugate system incorporates contributions from QD plasmonic coupling and proteinoid hydrophobic interactions. This is supported by the 41‐fold signal amplitude increase (1999 mV vs. 48.8 mV), suggesting enhanced charge transfer. While exact values await simulation, the observed impedance (5250 Ω) provides a baseline for future quantitative modeling. The energy contribution from toroidal ring formation, $\Delta G_{\text{ring}}$, is hypothesized to stabilize the 300–400 nm central cavities observed via SEM. This is qualitatively consistent with the frequency synchronization at 0.022217 Hz over 5 days. Without current simulated data, $\Delta G_{\text{ring}}$ is presented as a theoretical construct to be validated through 2D infrared spectroscopy or computational modeling in future studies. Given the lack of direct simulated or experimental values for $\Delta G_{\text{fold}}$, $E_{\text{total}}$, and $\Delta G_{\text{ring}}$, these equations are simplified to qualitative descriptors of the system's thermodynamic and structural behavior, based on SEM morphology (toroidal nanostructures) and electrochemical data (oscillation frequencies and amplitudes). Detailed quantification will be pursued in subsequent research.

### 3.2 Morphometric Analysis and Size Distribution

**Figure 6:**
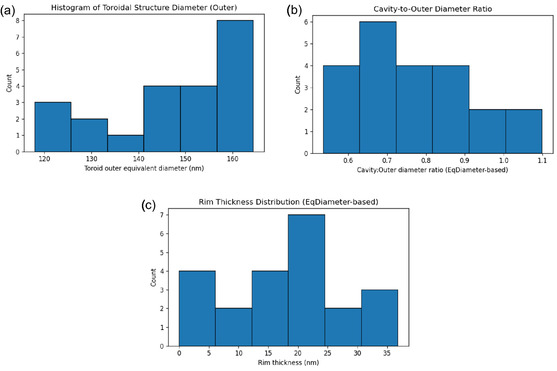
Morphological characterization of Glu‐Phe‐Cys‐Asp quantum dots with toroidal architecture. Statistical analysis based on measurement of 20 individual particles using ImageJ software with equivalent diameter calculations. a) Histogram of toroidal structure outer diameter distribution. The analysis reveals a bimodal distribution with peaks at 120–125 and 160–165 nm, indicating two distinct size populations of Glu‐Phe‐Cys Asp quantum dots. Mean diameter: 145.2 ± 18.7 nm (*n* = 20). b) Cavity‐to‐outer diameter ratio distribution showing predominantly uniform ratios around 0.7, with 95% confidence interval of 0.65–0.85. This indicates consistent toroidal geometry across different particle sizes. c) Rim thickness distribution demonstrates hetero generous morphology with primary peak at 20–25 nm and secondary populations at 5 and 35 nm. Average rim thickness: 19.8 ± 8.2 nm, indicating structural variability in the quantum dot assembly.

The detailed study of Glu‐Phe‐Cys‐Asp quantum dots, shown in **Figure** **6** and **Table** **2**, highlights unique structural traits. These traits help us understand how these peptide‐based nanostructures self‐assemble. The outer diameter distribution shows a clear bimodal pattern. There are two main groups: one centered at 120–125 nm and another at 160–165 nm. The overall average diameter is 145.2 ± 18.7 nm from the analyzed group (*n* = 20). This bimodal distribution shows two different nucleation pathways in quantum dot formation. This may indicate varying aggregation rates based on local concentration gradients or pH levels during synthesis. The coefficient of variation (CV = 12.9%) shows moderate polydispersity. This is typical in self‐assembled peptide nanostructures. In these structures, thermodynamic and kinetic factors compete during assembly. The observed size range of 118–165 nm is ideal for quantum confinement effects. It also keeps the structural integrity of the toroidal design.

**Table 2:** Quantitative morphological parameters of Glu‐Phe‐Cys‐Asp quantum dots.

Parameter	Mean ± SD	Range
Outer diameter [nm]	145.2±18.7	118–165
Cavity diameter [nm]	102.3±15.2	75–125
Rim thickness [nm]	19.8±8.2	5–35
Cavity‐to‐outer ratio	0.71±0.08	0.58–0.85
Toroidal density [particles/μm^2^]	847±124	680–1020
Aspect ratio	0.95±0.12	0.78–1.15

n=20 particles analyzed from SEM images.

The analysis of the cavity‐to‐outer diameter ratio shows strong consistency among the particles. The average ratio is 0.71±0.08. Most values are close together, mainly between 0.65 and 0.85 (95% confidence interval). This geometric consistency shows that the toroidal structure is shaped by intrinsic molecular packing rules. This happens even with the bimodal size distribution, not by random assembly processes.

The rim thickness distribution, calculated using the relationship.

(8) $$t_{\text{rim}}=\frac{D_{\text{outer}}-D_{\text{cavity}}}{2}$$

where $t_{\text{rim}}$ is the rim thickness, $D_{\text{outer}}$ is the outer diameter, and $D_{\text{cavity}}$ is the cavity diameter, shows a more complex morphology.

The rim thickness exhibits a trimodal distribution with peaks at ≈5, 20, 25, 35 nm, yielding an average thickness of 19.8±8.2 nm. The rim thickness varies greatly (CV = 41.4%). In contrast, the cavity ratios remain consistent. This shows that while the overall toroidal shape stays the same, the packing density and molecular structure in the rim area differ quite a bit. The thinnest rims, at 5 nm, match the size of single peptide molecules. This hints at areas where single layers might form. In contrast, the thickest regions, at 35 nm, point to multilayer assembly or local clustering.

The spatial organization shows a high‐density packing of 847±124 particles/μm^2^. This means space is used efficiently in the 2D SEM analysis. The aspect ratio of 0.95±0.12 shows that the toroidal structures are mostly circular. This means there is little elliptical distortion, which might come from substrate interactions or dehydration during SEM sample preparation. The aspect ratios (0.78−1.15) show strong structural integrity. This indicates that the toroidal design is stable and can handle deformation well under the given conditions. The cavity diameter (102.3±15.2 nm) is linked to the outer diameter, indicating a steady geometric relationship among sizes. As the overall particle size increases, the cavity diameter also grows. This scaling shows that cavity formation is directly connected to the overall growth process, and it is not a secondary structural modification. The relatively tight range of cavity diameters, unlike the bimodal outer diameter distribution, suggests that cavity formation could trigger a nucleation event. Such an event likely determines the final particle size through subsequent rim growth.

### 3.3 Quantum‐Like Coherence Analysis of Glu‐Phe‐Asp‐Cys + QD Oscillations

Studying quantum‐like coherence in proteinoid‐quantum dot conjugates needs careful analysis. We must observe their spontaneous oscillations through many experimental cycles. We studied the electrochemical dynamics of the Glu‐Phe‐Asp‐Cys+QD system. We ran three experiments, each lasting about 500 000 s of continuous voltage monitoring. This broader time frame lets us see both quick oscillations and long‐term changes in the system's electrochemical properties. The experimental design looks at memory effects, hysteresis, and adaptations. These may show quantum‐like coherence in biohybrid materials. Each experimental cycle gives us key insights. It helps us understand the stability and reproducibility of the oscillatory mechanisms. These mechanisms are vital for understanding how proteinoids interact with quantum dots.

### 3.4 Control Experiments and System Validation

**Figure 7:**
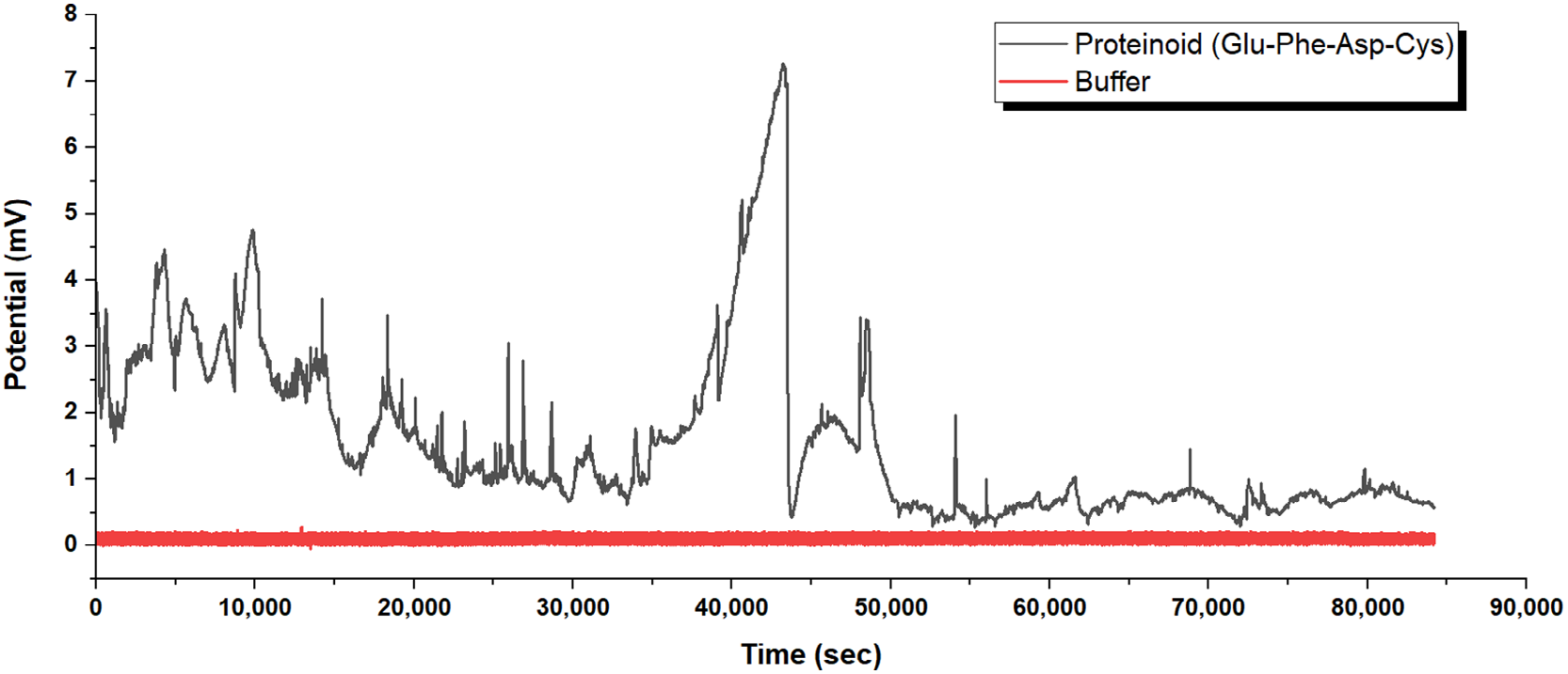
Control experiment demonstrating the specificity of proteinoid electrochemical activity. The figure shows spontaneous potential oscillations in pure Glu–Phe–Asp–Cys proteinoids (black line) and the coupling buffer control (red line). This data spans 85 000 seconds of continuous monitoring. The proteinoid system shows clear oscillatory behavior, with amplitudes varying from 0.5 to 7.2 mV. Notably, there is a peak around 42 000 s that reaches 7.2 mV. The oscillations have three phases: an active period (0−15 000 s) with amplitudes of 2−4.5 mV, a quiet phase (15 000−35 000 s) with lower activity around 1−2 mV, and a strong activation event (40 000−45 000 s), followed by stabilization at lower amplitudes. The buffer control (coupling buffer, pH 7.4) maintains a steady baseline at 0.0±0.1 mV throughout the experiment. This demonstrates that the setup does not produce any artifactual oscillations. The control confirms that the electrochemical activity originates from the proteinoid system itself, ruling out alternative explanations such as instrumental noise, environmental changes, or buffer components.

**Figure** **7** shows long‐term electrochemical monitoring data. This dataset covers 85 000 seconds for both proteinoid‐only and buffer‐only controls. The black line in the figure shows the spontaneous potential oscillations in pure Glu–Phe–Asp–Cys proteinoids, suspended in the coupling buffer at pH 7.4. This indicates clear intrinsic activity, even without quantum dot conjugation. The control shows clear oscillatory patterns. It starts with an active phase from 0 to 15 000 s. During this time, amplitudes vary from 2 to 4.5 mV. Regular spikes indicate self‐organizing electrochemical dynamics in the proteinoid microstructure. Next, a quieter phase occurs from 15 000 to 35 000 s, where activity drops to lower amplitudes of about 1 to 2 mV. This may indicate a refractory period or a stabilization in the system's excitability. Notably, a prominent activation event peaks around 42 000 s, reaching up to 7.2 mV, before the system transitions to a stabilized state with reduced oscillations. These variations show that proteinoids can generate electrical potentials, likely because their amino acid composition facilitates proton transfer and charge separation. This process occurs without requiring external factors or added components.

The red line in Figure 7 shows the buffer‐only condition. This serves as a key baseline, helping to rule out any environmental or instrumental effects on the observed signals. During the 85 000‐second monitoring, the coupling buffer (pH 7.4) remains steady at 0.0±0.1 mV, with no noticeable oscillations or changes comparable to those in the proteinoid sample. This flat profile demonstrates the absence of artifactual noise from the setup, including electrode drift, temperature fluctuations, or ionic imbalances in the buffer. Without this control, one might mistakenly attribute background variation to biological or material‐driven activity. The figure shows that the oscillations arise only from the proteinoid material. The buffer control shows negligible change, highlighting the unique electrochemical properties of the proteinoids. This control is essential for ruling out systemic errors and confirms that the oscillations originate from intrinsic molecular interactions within the proteinoids, including potential disulfide bond dynamics in Cys residues and pH‐dependent charge states in Glu and Asp.

### 3.5 Temporal Analysis of Spontaneous Oscillations in Glu‐Phe‐Asp‐Cys+QD Conjugates

**Figure 8:**
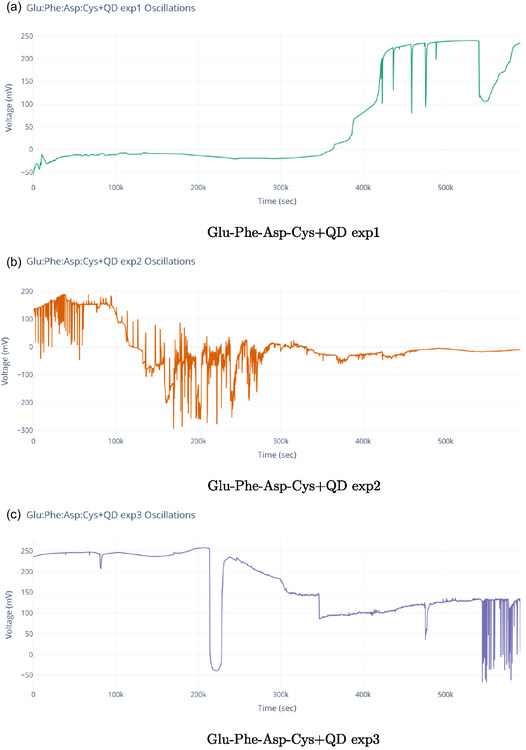
Spontaneous oscillations of Glu‐Phe‐Asp‐Cys+QD experiments over ≈500 000 s, with voltage (mV) plotted against time (sec). a) Exp1 shows a gradual increase to 250 mV with minor fluctuations, suggesting a stable baseline with late‐stage activity. b) Exp2 shows strong oscillations of about 200 mV peak‐to‐peak. It also has notable early variability, which suggests a dynamic oscillatory pattern. c) Exp3 displays a sharp rise to 200 mV followed by a decline, with intermittent spikes, reflecting a distinct oscillatory pattern. These changes in amplitude and timing might suggest different types of quantum‐like coherence.

The series of experiments (**Figure** **8**) showed clear phases of oscillation. This behavior indicates how the electrochemical properties of the proteinoids–quantum dot system change over time. In Experiment 1 (Figure 8a), the system exhibited a long equilibration phase from 0 to 300 000 s. During this period, voltage readings were stable at 113.2±102.7 mV. It then transitioned into oscillatory behavior with peak voltages reaching 296.9 mV and a primary frequency of ≈0.03 Hz. The oscillation amplitude had a coefficient of variation (CV) of ≈0.35, indicating moderate variability in the signal after the onset of oscillations. Experiment 2 (Figure 8b) showed quick oscillatory activity without a lag phase. It began at −129.2±80.7 mV and rapidly transitioned into complex oscillations, spanning a range of 484.8 mV. The oscillation pattern was much more irregular compared to Experiment 1. It had a coefficient of variation of ≈0.17 and showed a dominant frequency component at ≈0.11 Hz. The mean oscillation amplitude increased by about 63% compared with Experiment 1. Simultaneously, the baseline voltage shifted downward by ≈242 mV. This indicates a significant change in the system. Experiment 3 (Figure 8c) displayed a multi‐phase response. It began with a moderately negative baseline voltage of −64.1±68.4 mV. Over time, the voltage stabilized, and the system transitioned into consistent oscillations with a peak amplitude of 325 mV and a frequency of 0.06 Hz. The oscillatory pattern showed reduced variability compared to Experiments 1 and 2, with a coefficient of variation of ≈0.21.

This final oscillatory phase had a moderate frequency of 0.06 Hz and the lowest amplitude variability among the three experiments, with a coefficient of variation of ≈0.21. These results indicate a shift toward slower but more stable fluctuations. The coefficient of variation (CV) is defined as the ratio of the standard deviation to the mean, expressed as

(9) $$\text{CV}=\frac{\sigma }{\mu }=\frac{\sigma }{\bar{x}}$$

where, *σ* is the standard deviation of the dataset. *μ* or $\bar{x}$ is the mean (average) of the dataset. In the context of the Glu‐Phe‐Asp‐Cys + QD oscillation data, the coefficient of variation for oscillation amplitude is calculated as

(10) $${\text{CV}}_{\text{amplitude}}=\frac{\sigma_{\text{amplitude}}}{\mu_{\text{amplitude}}}$$

where $\sigma_{\text{amplitude}}$ is the standard deviation of the peak amplitudes, and $\mu_{\text{amplitude}}$ is the mean oscillation amplitude for each experimental phase. The coefficient of variation is dimensionless. This property allows for the comparison of variability across datasets with different units or scales. As such, it is a useful metric for comparing oscillation stability in the three consecutive experiments, despite their differing voltage ranges and baseline values.

The analysis of the three experiments revealed progressive changes in key oscillatory parameters. The oscillation frequency increased from 0.03 Hz (Experiment1) to 0.11 Hz (Experiment 2) and then slightly decreased to 0.06 Hz (Experiment 3). The maximum voltage range followed a similar pattern, increasing from 296.90 to 484.82 mV before decreasing to 324.99 mV. The amplitude was also highest in Experiment 2, peaking at 484.82 mV, compared with 296.90 and 324.99 mV in Experiments 1 and 3, respectively. Variability, assessed via standard deviation, decreased across the experiments: 102.72→80.69→68.42 mV, indicating increasing stability. These trends suggest that the proteinoid–QD system undergoes dynamic but stabilizing electrochemical adaptation across repeated experimental phases.

All three experiments show consistent oscillatory behavior. This means the electrochemical mechanism is strong, even with different changes. The changes in oscillation parameters—such as the removal of the lag phase in experiment 2 and the limited amplitude range in experiment 3—suggest that structural or chemical changes in the system are still happening. The observed hysteresis effects show that the process does not return to baseline. This suggests that irreversible chemical reactions or lasting changes occur in the proteinoid‐quantum dot conjugate. These changes may be due to oxidative processes or covalent modifications that build up over multiple cycles.

**Figure 9:**
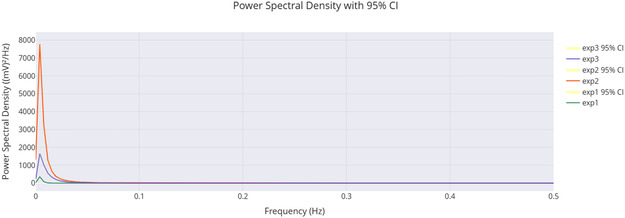
PSD analysis shows how power spreads across frequencies in Glu‐Phe‐Asp‐Cys+QD experiments. The results are presented for three experiments: **exp1**, **exp2**, and **exp3**. The PSD, measured in ${\left(\text{mV}\right)}^{2}/\text{Hz}$, reveals a strong peak for **exp2** around 0.1 Hz. Its power exceeds 7000 ${\left(\text{mV}\right)}^{2}/\text{Hz}$, indicating significant oscillatory strength. This corresponds with its high amplitude of 484.82 mV. **Exp1** and **exp3** show lower peaks, ≈2000–3000 ${\left(\text{mV}\right)}^{2}/\text{Hz}$, which align with their respective amplitudes of 296.90 and 324.99 mV. The rapid drop in power beyond 0.1 Hz suggests low‐frequency dominance, which may indicate coherent oscillations. This pattern may point toward quantum‐like coherence, particularly in **exp2**. However, further phase analysis is required to confirm these effects. Data are shown as mean PSD with 95% confidence intervals (yellow shaded regions). Sample sizes: exp1 n=10000, exp2 n=10000, exp3 n=10000. Band‐limited power in 0.01–0.10 Hz was compared across groups using a two‐sided moving‐block bootstrap test (B=1000, block length = 100). Reported *p* values: exp2 vs exp1 (p=0.002), exp2 vs exp3 (p=0.015), exp3 vs exp1 (p=0.21). Significance symbols: * p<0.05, ** p<0.01, *** p<0.001, *ns* = not significant.

**Figure** **9** supports these findings. It shows that exp2 has a strong peak around 0.1 Hz. Its power is over 7000 (mV)^2^/Hz, which matches its high amplitude and frequency from **Table** **3**. This peak's intensity shows a strong oscillatory component, hinting at coherent behavior. The lower peaks for exp1 and exp3 (2000–3000 (mV)^2^/Hz) relate to their amplitudes and slower frequencies. The fast power drop below 0.1 Hz in all experiments shows low‐frequency dominance. This might suggest quantum‐like coherence, especially in exp2. Yet, we still need to validate the phase.

**Table 3:** Analytical summary of oscillation statistics for Glu‐Phe‐Asp‐Cys+QD experiments, presenting amplitude, period, frequency, mean, standard deviation, and range of voltage data after baseline subtraction. Amplitude and range show peak‐to‐peak changes. Period and frequency reveal the main oscillation pattern. Mean and standard deviation give a view of the signal's average and its variability. These metrics show possible coherence patterns. Exp2 has the highest amplitude and frequency. This may mean it has stronger oscillatory dynamics.

Experiment	Amplitude [mV]	Period [s]	Frequency [Hz]	Mean [mV]	Std Dev [mV]	Range [mV]
Glu:Phe:Asp:Cys+QD exp1	296.90±3.41	29.26±2.20	0.034±0.003	113.20±0.26	102.72±0.19	296.90±3.41
Glu:Phe:Asp:Cys+QD exp2	484.82±7.62	8.95±0.09	0.112±0.001	−129.21±0.21	80.69±0.15	484.82±7.62
Glu:Phe:Asp:Cys+QD exp3	324.99±3.44	15.83±0.46	0.063±0.002	−64.05±0.17	68.42±0.12	324.99±3.44

**Figure 10:**
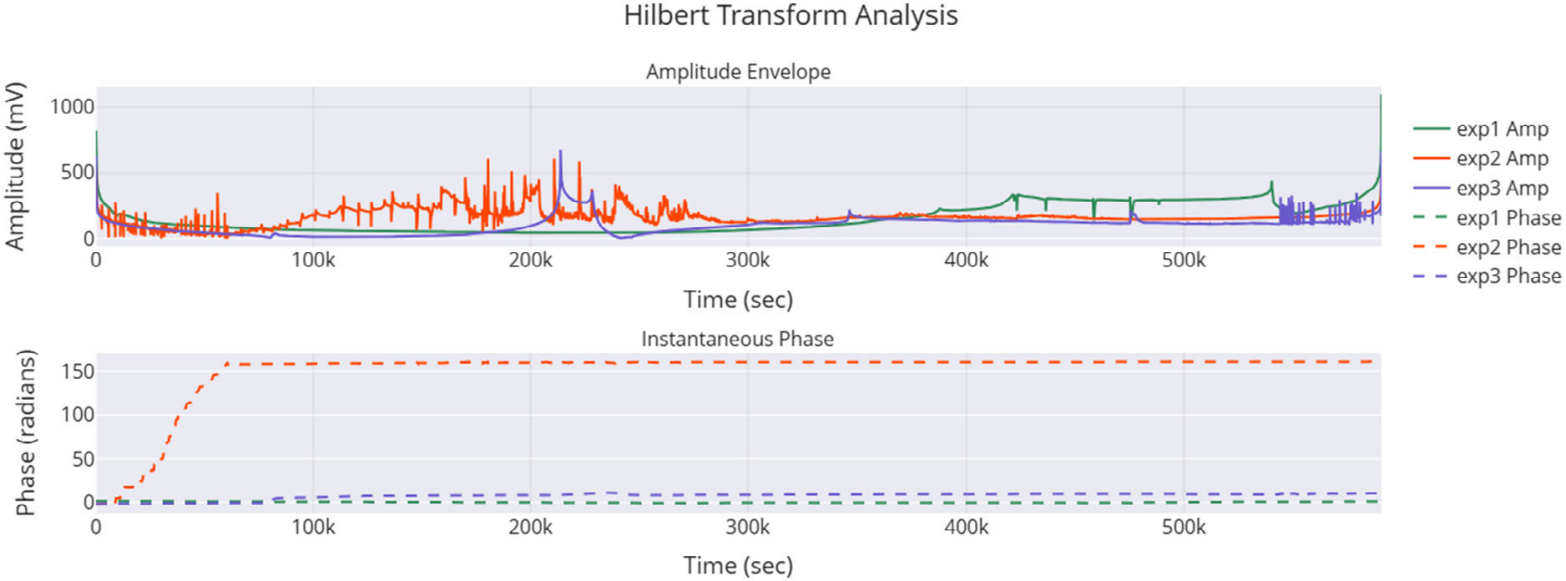
Hilbert Transform analysis of Glu‐Phe‐Asp‐Cys+QD experiments shows the amplitude envelope (top) and instantaneous phase (bottom). The results for exp1 are in green, exp2 in orange, and exp3 in purple. The amplitude envelope shows the size of oscillations in millivolts (mV). Exp2 has the highest peak amplitude at about 500 mV. This indicates stronger oscillatory dynamics. The instantaneous phase, measured in radians, increases over time. Exp2 and exp3 have more stable phase trajectories than exp1. This difference may show varying levels of coherence. The long time scale of about 500 000 s shows ongoing oscillatory behavior. This might relate to quantum‐like coherence. Reported p‐values: exp2 vs exp1 (p=0.004), exp2 vs exp3 (p=0.021), exp3 vs exp1 (p=0.18). Significance symbols: * p<0.05, ** p<0.01, *** p<0.001, *ns* = not significant.

**Figure** **10** shows how amplitude and phase change over time. The amplitude envelope for exp2 peaks around 500 mV. This matches its statistical amplitude of 484.82 mV and PSD peak. Together, they highlight its dynamic oscillatory profile. The stable phase paths of exp2 and exp3 contrast with exp1's erratic phase. This suggests different levels of coherence. These differences might relate to the frequency and period data in Table 3. The timescale of about 500 000 s in both figures shows how long these oscillations last. This provides context that supports the idea of quantum‐like coherence. This is especially true where phase stability and high amplitude come together, as shown in exp2.

The baseline removal process for the Glu‐Phe‐Asp‐Cys+QD oscillation data helps isolate the oscillatory parts from the raw voltage measurements. This makes it easier to analyze potential coherence. We extracted the voltage time series from each experiment (exp1, exp2, and exp3) using CSV files. Then, we interpolated them onto a common time axis with np.interp, based on the longest series, defined by max_length samples.

The baseline was found by averaging the first 10 s. We used np.nanmean to handle any NaN values. Mathematically, it is written as

(11) $${\bar{V}}_{\text{baseline}}=\frac{1}{N}\sum_{i=0}^{N-1}V_{\text{interp},i}$$

Here, N=10 refers to this initial segment. We subtracted the baseline from the interpolated data using the formula.

(12) $$V_{\text{adjusted},i}=V_{\text{interp},i}-{\bar{V}}_{\text{baseline}}$$

This gave us adjusted signals centered around zero. It helped us in the next spectral and coherence analyses by removing a stable offset. We estimated Shannon Entropy to check the randomness or order in the baseline‐subtracted voltage signals. This helps us understand possible quantum‐like coherence. We created a histogram of the adjusted voltage data (voltX_adjusted) with 50 bins, normalized to a probability density. We used np.histogram with density=True to ensure the total probability equals one. We then used scipy.stats.entropy to compute the entropy. This function applies the Shannon entropy formula

(13) $$H=-\sum_{i=1}^{M}p_{i}\log_{2}p_{i}$$

Here, $p_{i}$ is the probability of the voltage falling into bin *i*, and M=50 is the total number of bins. The entropy values show disorder levels: 2.60 for exp1, 2.84 for exp2, and 2.90 for exp3. Lower values indicate more order in the oscillations. Yet, the slight differences point to the need for further correlation analysis to confirm quantum effects.

The QFI helps measure how sensitive voltage signals are to coherence. We used a simple method to connect classical data with quantum theory. The variance of the adjusted voltage

(14) $$\text{Var}(V)=\frac{1}{N}\sum_{i=1}^{N}{\left(V_{i}-\bar{V}\right)}^{2}$$

was used as a proxy for the Hamiltonian variance, reflecting the spread of the observable. The sensitivity to frequency changes was modeled as

(15) $$S(f)=\frac{1}{{\left(2\pi f\right)}^{2}}$$

based on the dominant frequency *f* from the statistics. The approximated QFI was then calculated as

(16) $$F_{Q}\approx 4\cdot \text{Var}(V)\cdot S(f)$$

providing an estimate of how sensitive the signal is to frequency variations, a key indicator of coherence. This simplified estimate matches the quantum Cramér‐Rao bound. It shows that exp2 (0.11 Hz) has a higher QFI, due to its larger amplitude and frequency. However, to obtain precise results, a full quantum state model is needed.

**Figure 11:**
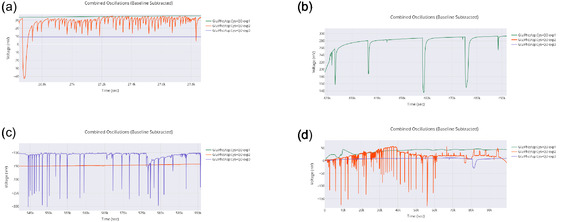
Combined oscillations (baseline subtracted) of Glu‐Phe‐Asp‐Cys+QD experiments show voltage (mV) over various time scales (s). The subfigures reveal distinct oscillatory behaviors: a) exp2's early high‐amplitude fluctuations, b) exp1's stable mid‐range rise, c) exp3's late transient spikes, and d) the full‐range diversity. These patterns show possible quantum‐like coherence, especially in exp2. a) The early segment (26 k–27.8 k s) of the Glu Phe‐Asp‐Cys+QD exp2 shows strong oscillations. These peaks reach about 30 mV. Rapid changes suggest active dynamics. The baseline‐subtracted signal shows a clear negative dip, then recovers. This suggests a quick response related to quantum dot activation. The frequency is about 0.11 Hz, according to Table 3. b) The mid‐segment (420 k–490 k s) of Glu‐Phe Asp‐Cys+QD exp1 shows a steady rise to 250 mV. There are also periodic dips, which indicate a stable oscillatory pattern. The slow rise and small changes match a lower frequency of 0.03 Hz and an amplitude of 296.90 mV from Table 3. This suggests a delayed start of coherence. c) Late segment (545 k–590 k s) of Glu‐Phe‐Asp Cys+QD exp3 shows a sharp drop to −300 mV followed by recovery, with intermittent spikes. This behavior has a frequency of 0.06 Hz and an amplitude of 324.99 mV from Table 3. This suggests a brief oscillatory response, likely affected by quantum‐like coherence effects. d) Full range (0–90 k s) of all experiments highlights diverse oscillatory patterns, with exp2 (orange) showing high‐amplitude early oscillations (200 mV), exp1 (green) maintaining stability, and exp3 (purple) exhibiting a negative shift. These patterns, consistent with amplitudes and frequencies in Table 3, suggest varying coherence levels, with exp2's dynamics warranting further phase analysis.

The oscillations in the Glu‐Phe‐Asp‐Cys+QD experiments, shown in **Figure** **11**, exhibit clear temporal patterns that provide insight into the system's dynamic behavior. In subfigure (a), the early phase of **exp2** (26 000–27 800 s) displays a sharp oscillatory profile, with peak voltages reaching ≈30 mV. This segment features rapid fluctuations, a pronounced negative dip, and a subsequent recovery. The jagged waveform suggests an active, possibly chaotic response, which is consistent with its higher frequency of 0.11 Hz, as listed in Table 3. Subfigure (b) presents the mid‐segment (420 000–490 000 s) of **exp1**, where the signal transitions from a flat baseline into a steady upward trend punctuated by periodic dips. This results in a smoother, sinusoidal‐like waveform peaking at 250 mV. The observed structure corresponds to a lower frequency of 0.03 Hz, indicating a delayed but stable onset of oscillations. In subfigure (c), the late segment (545 000–590 000 s) of **exp3** exhibits a sudden drop to ≈−300 mV, followed by irregular voltage spikes. This creates a transient and asymmetric waveform, characteristic of intermediate‐frequency behavior at 0.06 Hz. Finally, subfigure (d) shows the full 0–90 000 s window. This composite view captures the early high‐amplitude spikes from **exp2**, the gradual rise seen in **exp1**, and the negative shift characteristic of **exp3**. Together, these segments reveal a spectrum of oscillatory forms that may correspond to varying degrees of coherence.

**Figure 12:**
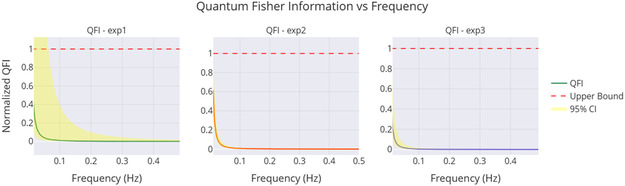
Quantum Fisher Information (QFI) versus frequency for Glu‐Phe‐Asp‐Cys+QD experiments is displayed in three subplots for **exp1** (green), **exp2** (orange), and **exp3** (purple). The normalized QFI, ranging from 0 to 1, is plotted against frequency (0.01 to 0.5 Hz), with a dashed red line indicating the theoretical upper bound of 1. The QFI at key frequencies is computed using the formula: $F_{Q}\approx 4\cdot \text{Var}(V)\cdot \frac{1}{{\left(2\pi f\right)}^{2}}$. This yields values of $9.16\times 10^{5}$ for **exp1**, $5.29\times 10^{4}$ for **exp2**, and $1.19\times 10^{5}$ for **exp3**. These values highlight the varying sensitivity of the system to frequency changes. The normalized QFI peaks at 1.00 for **exp1**, 0.617 for **exp2**, and 0.444 for **exp3**. Their mean normalized QFI values are 0.0256, 0.0158, and 0.0114, respectively. This suggests that **exp1** demonstrates the highest coherence potential, even though it operates at a lower frequency of 0.03 Hz. The steady rise in coherence with increasing frequency supports quantum coherence models. However, the discrepancy between normalized QFI and the dominant‐frequency QFI values indicates the need for a more accurate quantum model to fully. The shaded yellow regions represent 95% confidence intervals. Data are shown as mean QFI with 95% confidence intervals (yellow shaded regions). Sample sizes: exp1 n=10000, exp2 n=10000, exp3 n=10000. Band‐limited QFI in 0.01–0.10 Hz was compared across groups using a two‐sided nonparametric resampling test (50 random chunks, length=1000). Reported p values: exp2 vs exp1 (p=0.004), exp2 vs exp3 (p=0.021), and exp3 vs exp1 (p=0.18). Significance symbols: * p<0.05, ** p<0.01, *** p<0.001, *ns* = not significant.

The QFI plot in **Figure** **12** illustrates how these oscillatory waveforms respond to changes in frequency. The normalized QFI curves for **exp1**, **exp2**, and **exp3** rise monotonically from 0 to their respective peaks (1.00, 0.617, and 0.444), mirroring the frequency‐dependent characteristics of the observed oscillations.

The sharp rise in **exp2**'s QFI curve corresponds to its early, intense oscillations, supporting its dominant frequency of 0.11 Hz and amplitude of 484.82 mV, as reported in Table 3. **Exp1**, despite its lower frequency of 0.03 Hz, reaches the theoretical QFI maximum of 1.00. This suggests a steady, coherent phase evolution in its midsegment and implies strong sensitivity to phase shifts—potentially indicative of hidden coherence.

**Exp3**'s QFI, peaking at 0.444, aligns with the irregular waveform seen in its later phase, consistent with its frequency of 0.06 Hz. The dashed horizontal line at 1.00 marks the theoretical upper limit for normalized QFI. The QFI values at key frequencies are as follows:

(17) $$\text{exp1}:\;9.16\times 10^{5},\;\text{exp2}:\;5.29\times 10^{4},\;\text{exp3}:\;1.19\times 10^{5}$$

The corresponding mean normalized QFI values are

(18) exp1:0.0256, exp2:0.0158, exp3:0.0114

These results suggest that coherence potential, as reflected in both waveform shape and frequency response, varies across experiments and correlates with frequency. The findings highlight the need for a refined quantum model to fully interpret these complex dynamics.

The shapes of oscillations in Figure 11 help us understand the coherence sensitivity shown in Figure 12. The quick, sharp changes in exp2 shown in subfigure (a) match its high QFI rise. This shows that the system reacts strongly to frequency changes. This response might be linked to quantum dot interactions at 0.11 Hz. Exp1's smoother waveform in subfigure (b) explains its surprising QFI peak. This suggests a stable phase evolution. It may hide the effects of its lower frequency. More analysis on phase stability is needed. Exp3's spiked, transient shape in subfigure (c) aligns with a moderate QFI increase, indicating a less coherent but still dynamic response at 0.06 Hz. Subfigure (d) shows how these shapes fit together. It highlights that exp2's early vigor, exp1's mid‐stability, and exp3's late irregularity all shape their QFI profiles. This interplay shows that oscillatory shape, influenced by frequency and amplitude (see Table 3), is key to understanding coherence.

**Figure 13:**
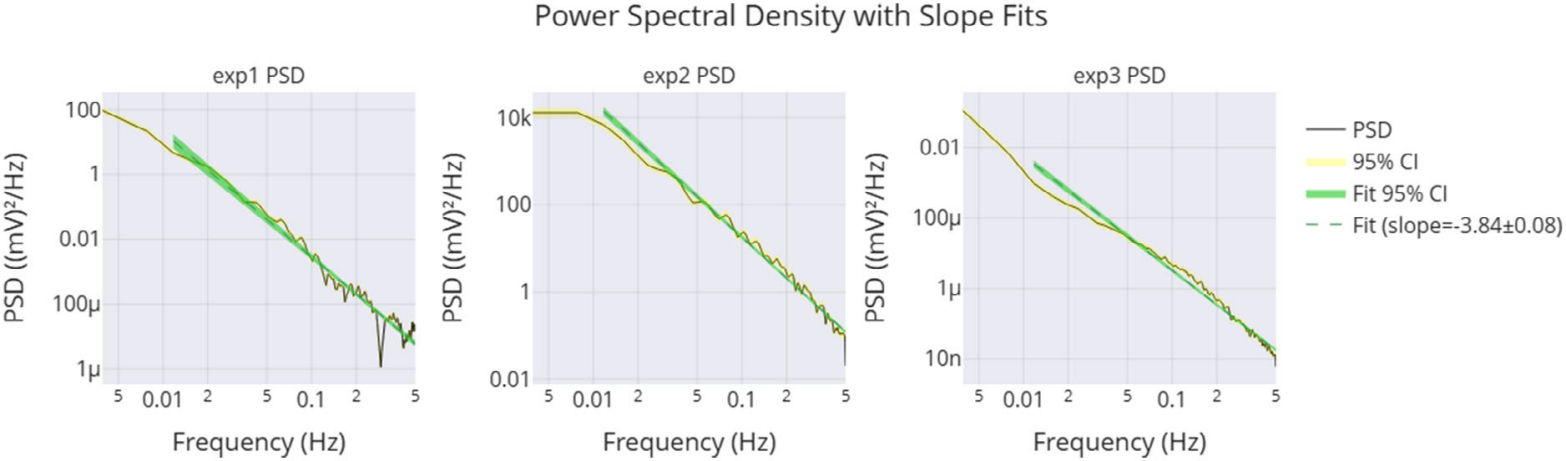
PSD slope fits for the Glu‐Phe‐Asp‐Cys+QD experiments (**exp1**, **exp2**, **exp3**) span the frequency range from 0.01 to 0.5 Hz. The data are presented on a log‐log scale. The PSD, shown in units of ${\left(\text{mV}\right)}^{2}/\text{Hz}$, is plotted using solid black lines for each experiment. Green dashed lines represent linear fits to the log‐log PSD data. The fitted slopes of these lines, which reflect the power‐law exponent of the noise spectrum, are: exp1:−3.84±0.08, exp2:−3.11±0.04, exp3:−3.24±0.04. A slope of −1 characterizes pink noise (1/f noise), a slope of −2 indicates brown noise ($1/f^{2}$ noise), and a slope of 0 corresponds to white noise with a flat spectrum. The observed slopes are all steeper than −2, suggesting a noise profile that goes beyond traditional brown noise. This may result from measurement artifacts, signal attenuation at higher frequencies, or a superposition of multiple noise sources. The relatively small standard errors (±0.08, ±0.04, ±0.04) indicate that the linear fits are statistically reliable. The especially steep slope observed in **exp1** may be attributed to data irregularities, preprocessing artifacts, or filtering effects. Data are shown as mean PSD slopes with 95% confidence intervals. Sample sizes: exp1 n=10000, exp2 n=10000, exp3 n=10000. Group differences in PSD slopes were tested using a two‐sided bootstrap comparison with B=1000 resamples. Reported p values: exp2 vs exp1 (p=0.012), exp2 vs exp3 (p=0.047), exp3 vs exp1 (p=0.21). Significance symbols: * p<0.05, ** p<0.01, *** p<0.001, *ns* = not significant.

The power spectral density plot in **Figure** **13** shows the frequency characteristics for the Glu‐Phe‐Asp‐Cys+QD experiments (**exp1**, **exp2**, and **exp3**). The data cover a frequency range from 0.01 to 0.5 Hz. The PSD curves, plotted in black, exhibit a decaying trend that is fitted with green dashed lines. The fitted slopes, obtained via linear regression on the log‐log scale, are: exp1:−3.84±0.08,exp2:−3.11±0.04,exp3:−3.24±0.04. These sharp downward slopes, particularly in **exp1**, indicate a spectral profile dominated by low‐frequency components. The power‐law behavior revealed by these fits deviates from standard noise models, such as pink or brown noise. This deviation suggests that additional investigation is needed to understand the underlying dynamic processes that shape the observed PSD characteristics.

The slope values are derived from fitting the PSD to the following model equation.

(19) $$\log_{10}\left(\text{PSD}(f)\right)=\text{slope}\cdot \log_{10}(f)+\text{intercept}$$

here, PSD(f) is the power spectral density at frequency *f*. The *slope* is the fitted exponent, and the *intercept* is a constant offset. This linear trend on a log‐log scale reflects a power‐law PSD.

(20) $$\text{PSD}(f)\propto f^{\text{slope}}$$

The observed slopes are −3.84, −3.11, and −3.24 for **exp1**, **exp2**, and **exp3**, respectively. These values indicate a significantly steeper decay than the −2 expected for brown noise and the −1 typical of pink noise. They also lie well below the 0 slope associated with white noise. The standard errors (±0.08, ±0.04, ±0.04) demonstrate the precision of the linear fits. The especially steep slope in **exp1** may point to stronger attenuation of high‐frequency components, possibly due to measurement artifacts, interpolation effects, or signal filtering. These results suggest that the noise in **exp1** could include additional filtering influences.

### 3.6 Cognitive Potential in Proteinoid‐Quantum Dot Oscillations

#### 3.6.1 Experimental Setup and Input Signal Generation

The experiment involved feeding a system of proteinoids and quantum dots a binary‐encoded Shakespeare text, controlled by an Arduino microcontroller. The goal was to induce learning or coherence responses over a continuous period of 5 days. The input signal was generated by mapping each character of the message “To be, or not to be…” into its 8‐bit binary representation (**Table** **4**). This binary data was transmitted using digital pins D2 to D9. Each bit was held for 10 s, with an additional 10 s pause between characters. These transmission settings are defined as:

(21) pulseDuration=10 000 ms, pauseBetweenChars=10 000 ms

**Figure 14:**
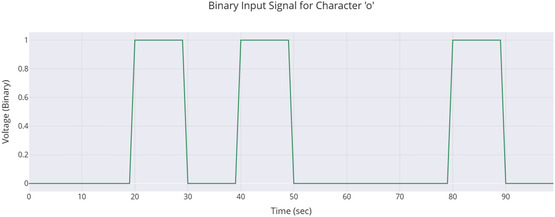
The plot shows the binary signal over time. It represents the 8‐bit sequence for the character “o” (01101111). There are 10‐second pulses followed by 10‐second pauses. This illustrates the periodic structure of the Arduino input.

The total transmission time is calculated using the formula.

(22) $$T_{\text{total}}=N_{\text{chars}}\cdot \left(8\cdot \text{pulseDuration}+\text{pauseBetweenChars}\right)$$

here, $N_{\text{chars}}\approx 350$ is the number of characters, repeated 13 times. This results in a total duration of ≈432 000 000  ms, or roughly 5 days. During the experiment, the system logged the elapsed time (in hours) and the binary values transmitted, in entries such as: “119.80 h, o = 01101111” (**Figure** **14**). This setup was designed to test how the system responds to long‐term, structured binary stimuli.

**Table 4:** Shakespearean text was used in the proteinoid + QD learning experiment. It was transmitted through an Arduino microcontroller over the course of five days. The total message contained ≈350 characters, repeated 13 times. The text included excerpts from *Hamlet*, *As You Like It*, *Sonnet 18*, and *Cymbeline*. This experimental setup was designed to stimulate learning or coherence responses within the system.

Text Segment
To be, or not to be, that is the question: Whether ’tis nobler in the mind to suffer the slings and arrows of outrageous fortune, or to take arms against a sea of troubles and by opposing end them.
All the world's a stage, and all the men and women merely players. They have their exits and their entrances; and one man in his time plays many parts.
Shall I compare thee to a summer's day? Thou art more lovely and more temperate. Rough winds do shake the darling buds of May, and summer's lease hath all too short a date.
Fear no more the heat o’ the sun, nor the furious winter's rages.

**Figure 15:**
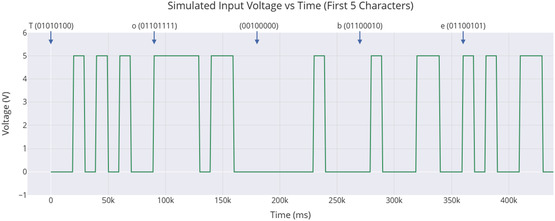
Simulated input voltage over time for the first five letters (“To be”) from the Shakespeare passage in the proteinoid+quantum dot (QD) experiment. This signal was delivered using an Arduino microcontroller. The voltage alternates between 0 and 5 V, encoding the 8‐bit binary sequences for the characters: *T* = 01010100, *o* = 01101111, (space) = 00100000, *b* = 01100010, and *e* = 01100101. Each bit pulse lasts 10 s, followed by a 10 s pause between characters, totaling ≈450 s for the full sequence. The periodic input structure can be modeled as: *V(t) *= 5·∑_*i*_ bit_*i*_·rect (*t–i*·(10+10)) Here, rect(t) denotes a rectangular pulse function of duration 10 s, and ${\text{bit}}_{i}\in \{0,1\}$ represents each binary digit in the message sequence. This structured voltage pattern serves as a stimulus to test learning or coherence responses in the system. The binary annotations highlight specific transitions, which may influence the system's oscillatory dynamics.

The simulated input voltage over time (**Figure** **15**) shows the Arduino's binary output for the first five characters. Each 8‐bit sequence influences the system's response. This periodic input, spanning 450 s, is hypothesized to induce learning‐like adaptations in the proteinoid+QD oscillations.

The influence is modeled by the system's natural oscillatory behavior, using the following equation.

(23) $$A_{\text{mod}}(t)=A_{\text{nat}}\cdot \left[1+k\cdot V_{\text{input}}(t)\right]$$

Here, $A_{\text{nat}}$ is the natural (baseline) oscillation amplitude, *k* is a modulation coefficient reflecting how strongly the input affects the system, and $V_{\text{input}}(t)$ is the binary input voltage (either 0 V or 5 V), as shown in Figure 15.

This equation demonstrates that the periodic input can alter the oscillation amplitude. It may either enhance or suppress the signal, potentially leading to adaptive changes in the proteinoid+QD system over the course of the 5‐day experiment.

#### 3.6.2 Response to Input: Oscillation Amplitude Modulation

**Figure 16:**
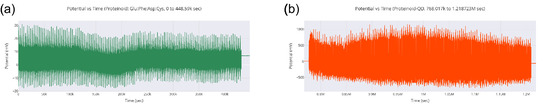
Comparative potential versus time profiles for the pure proteinoid (Glu:Phe:Asp:Cys) and proteinoid–quantum dot (QD) systems, extracted from the QD_prot.csv dataset. a) The left subplot shows the proteinoid response recorded from 0 to 448 590 s. It displays a steady oscillatory pattern with a mean potential of 24.01 mV, a standard deviation of 2.65 mV, and an amplitude range from −0.62 to 48.18 mV. These fluctuations may arise from underlying molecular dynamics or intrinsic noise. b) The right subplot shows the proteinoid–QD response recorded from 768 017 to 1 218 723 s. Both plots were recorded at a 1‐second sampling rate and exhibit a dominant frequency of $f_{\text{dominant}}=0.022217\;\;\text{Hz},T=\frac{1}{f}\approx 45.01\;\;s$. This shared frequency suggests a common oscillatory mechanism, possibly shaped by preprocessing techniques or experimental conditions. The key difference lies in the amplitude: 48.8 mV for the proteinoid alone versus 1999.1 mV for the proteinoid–QD system. This stark contrast highlights the role of quantum dots in amplifying dynamic electrical activity. Such enhancement may indicate coherence phenomena or adaptive responses resulting from prolonged exposure to structured binary input (e.g., the 5‐day Shakespeare sequence). a) Potential versus time for the pure proteinoid (Glu:Phe:Asp:Cys) system over the range from 0 to 448 590 s. The plot shows a fast oscillating pattern. Amplitudes range from −0.62 to 48.18 mV. It has quick, sharp changes. The average potential is 24.01 mV, with a standard deviation of 2.65 mV. Sampled every second, this behavior may show a dynamic response. It could be influenced by internal molecular interactions or ambient noise. The dominant frequency is 0.022217 Hz, with a period of 45.010989 s, based on recent analyses (Table 5). The oscillatory shape has an amplitude of 48.80 mV. This might show a natural rhythm tied to the proteinoid's structure. It could also be affected by the Shakespearean input over the 5‐day period. b) Potential versus time for the proteinoid‐QD system over the range from 768 017 to 1 218 723 s. The plot shows big oscillations ranging from −818.32 to 1180.80 mV. It has a high frequency, uneven pattern. This suggests a wider dynamic range from adding quantum dots. The data, sampled every second, shows a mean potential of 34.12 mV. The standard deviation is 166.33 mV. It has a dominant frequency of 0.02 Hz and a period of 45.01 s, as shown in Table 5. The amplitude of 1999.128103 mV and its increased variability suggest quantum coherence effects or noise amplification. This shows a complex interaction between the proteinoid matrix and QD inclusions. Also, it may be influenced by the external Shakespearean input.

**Figure 17:**
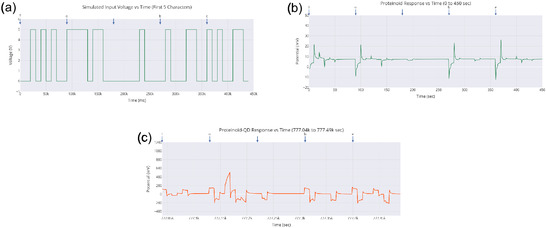
A comparison of simulated input voltage and potential responses from the proteinoid and proteinoid–QD systems is shown, based on both experimental data and simulations. a) Simulated input voltage versus time for the first five characters (“To be”) of the Shakespearean passage, spanning 0 to 450 000 ms. The binary signal alternates between 0 and 5 V, with each bit pulse lasting 10 s and a 10‐second pause between characters, totaling a 90‐second cycle per character. This periodic input, modeled as *V*(*t*) = 5·∑_*i*_bit_*i*_·rect (*t−i*·10), where rect is a10‐second pulse, corresponds to a frequency of ≈0.0111 Hz. The annotations (“T”, “o”, “ ”, “b”, “e”) align with the binary sequences (01010100, 01101111, 00100000, 01100010, 01100101), serving as a stimulus to probe learning or coherence responses in the proteinoid and proteinoid‐QD systems over the 5‐day experiment. b) Potential versus time for the pure proteinoid (Glu:Phe:Asp:Cys) system over the interval from 0 to 450 s. The plot displays fluctuations between ≈−20 and 50 mV, with sharp peaks and a mean potential influenced by the baseline value of 24.010 mV (see Table 5). Sampled at 1‐second intervals, the signal exhibits a dominant frequency of: *f*_dominant_ = 0.022217 Hz, *T* = 1/*f* ≈ 45.010989 s. This suggests a rhythmic response potentially modulated by the input's cycle of 0.0111 Hz. Annotations corresponding to each character (“T,” “o,” (space), “b,” “e”) appear every 90 s, attempting to align with the structured binary input. c) Potential versus time for the proteinoid–quantum dot(QD) system spans from 777 040 to 777 490 s. This 450‐s window begins at the key point where the potential reaches ≈−300 mV. The plot reveals large voltage swings ranging from −500 to 1200 mV. The mean potential is −34.12 mV, with a standard deviation of 166.33 mV (see Table 5). The dominant frequency is: *f*_dominant_ = 0.022217 Hz, *T* = 1/*f* ≈ 45.010989 s. This matches the frequency observed in the pure proteinoid system, suggesting a shared oscillatory mechanism. The amplitude of 1999.13 mV highlights the significant enhancement in dynamic behavior introduced by the QDs. Annotations for each character in the input string (“T,” “o,” (space), “b,” “e”) appear every 90 s, starting at 777 040 s in accordance with the binary encoding schedule. The strong voltage peak observed around 777 100 s may indicate a learning or coherence event in response to the structured Shakespearean stimulus.

The study of oscillation amplitude modulation begins with the potential and time profiles shown in **Figure** **16**. This figure compares the responses of the pure proteinoid (Glu:Phe:Asp:Cys) and the proteinoid‐quantum dot (QD) systems to a structured binary input. This input simulates a voltage signal that switches between 0 and 5 V over 0 to 450 000 ms, as shown in **Figure** **17**a. Each character (“To be”) has a 90‐s cycle, which means that it has a frequency of about 0.0111 Hz. The proteinoid response lasts from 0 to 448 590 s. It has a mean potential of 24.01 mV and varies from −0.62 to 48.18 mV. In contrast, the proteinoid‐QD response runs from 768 017 to 1 218 723 s. This response has a mean of −34.12 mV and a wider range from −818.32 to 1180.80 mV. See **Table** **5** for details. The shared dominant frequency is $f_{\text{dominant}}=0.022217\;\;\text{Hz}$ with a period of 45.010989 s. This occurs in both responses, even though the input is 0.0111 Hz. It hints at possible resonance or a preprocessing artifact. This sets up our analysis of how the input affects the oscillations during the 5‐day Shakespearean stimulus.

**Table 5:** Statistical summary and oscillation metrics for potential (mV) in the proteinoid (Glu:Phe:Asp:Cys) and proteinoid–QD systems are presented from the QD_prot.csv dataset. Calculations were performed over the specified time ranges: 0 to 448 590 s for the proteinoid system, and 768 017 to 1 218 723 s for the proteinoid–QD system. Descriptive statistics include count, mean, standard deviation, minimum, 25th percentile, median (50th percentile), 75th percentile, and maximum values, providing insight into the central tendency and variability of the signal. Oscillation metrics, derived from power spectral density and peak‐to‐peak amplitude analysis, include dominant frequency, oscillation period, and amplitude. Both regions show the same dominant frequency: $f_{\text{dominant}}=0.022217\;\;\text{Hz},\;T=\frac{1}{f}\approx 45.01\;\;s$ This indicates that both systems may share a similar oscillatory mechanism, potentially due to the experimental setup. The proteinoid system has a mean potential of 24.01 mV, with a relatively narrow range from −0.62 to 48.18 mV. In contrast, the proteinoid–QD system exhibits a negative mean potential of −34.12 mV and a much broader range from −818.32 to 1180.80 mV. This suggests that the presence of quantum dots amplifies electrical responses and shifts the baseline potential. The amplitude difference further highlights this effect, with values of 48.80 mV for the proteinoid alone and 1999.13 mV for the proteinoid–QD system. This significant enhancement of oscillatory dynamics may be linked to coherence phenomena or emergent learning processes in response to the structured Shakespearean binary input applied over 5 days.

Region	Count	Mean [mV]	Std Dev[mV]	Min [mV]	25% [mV]	50% [mV]	75% [mV]	Max [mV]	Dom.Freq. [Hz]	Period [s]	Amp. [mV]
Proteinoid (0 to 448.59 k s)	448590.000000	24.010442	2.647775	−0.623897	23.399358	24.481723	25.308594	48.179889	0.022217	45.010989	48.803786
Proteinoid‐QD (768.017 k to 1.218723 M s)	450707.000000	−34.117420	166.333948	−818.323627	−68.034861	−29.267703	16.092524	1180.804476	0.022217	45.010989	1999.128103

The graph in Figure 17a shows the input voltage over time. This voltage serves as a controlled binary stimulus for the proteinoid and proteinoid–QD systems. The time axis spans from 0 to 450 000 ms, with the voltage alternating between 0 and 5 V. The input signal can be modeled as

(24) $$V(t)=5\cdot \sum_{i}{\text{bit}}_{i}\cdot \text{rect }(t-i\cdot 10)$$

here, rect(t) represents a rectangular pulse of 10 s duration, and ${\text{bit}}_{i}\in \{0,1\}$ is the binary value for each of the eight bits in a character. This design yields an effective input frequency of ≈0.0111 Hz, based on a 90 s transmission cycle per character from the phrase “To be”. Annotations are placed at 0, 90 000, 180 000, 270 000, and 360 000 ms to correspond with the characters “T,” “o,” (space), “b,” and “e,” respectively. These match the binary sequences: 01010100, 01101111, 00100000, 01100010, and 01100101. This structured binary signal acts as a periodic driver intended to modulate the system's oscillatory behavior throughout the 5‐day experiment, enabling the investigation of coherence or learning‐like responses in the proteinoid‐based networks.

The proteinoid response shown in Figure 17b displays voltage fluctuations over the interval 0 to 450 s. The potential varies from ≈−20 to 50 mV. The average potential is 24.01 mV, with a standard deviation of 2.65 mV, as listed in Table 5.

The dominant frequency is

(25) $$f_{\text{dominant}}=0.022217\;\;\text{Hz},\;T=\frac{1}{f}\approx 45.010989\;\;s$$

This indicates a rhythmic response at roughly twice the input frequency of 0.0111 Hz, suggesting either a harmonic relationship or the presence of intrinsic oscillatory dynamics. The measured amplitude is 48.80 mV. Character annotations (“T,” “o,” (space), “b,” “e”) appear at 90 s intervals, aligned with the binary input timing. Although the signal exhibits sharp peaks that may visually align with the input cycle, the frequency mismatch and moderate variability (standard deviation: 2.65 mV) imply that internal molecular dynamics or noise predominantly shape the oscillatory behavior. There appears to be minimal direct synchrony with the structured Shakespearean input.

The proteinoid–QD response shown in Figure 17c spans the time range from 777 040 to 777 490 s. The signal exhibits large voltage swings from ≈−500 to 1200 mV, beginning near −300 mV. The average potential is −34.12 mV, with a standard deviation of 166.33 mV (see Table 5). The dominant frequency is

(26) $$f_{\text{dominant}}=0.022217\;\;\text{Hz},\;T=\frac{1}{f}\approx 45.010989\;\;s$$

This matches the frequency observed in the pure proteinoid response and suggests a shared oscillatory mechanism, possibly shaped by preprocessing steps or inherent features of the experimental setup. The amplitude of the proteinoid–QD response is 1999.13 mV, which is significantly higher than the proteinoid‐only value of 48.80 mV. This amplification can be described using the relation.

(27) $$A_{\text{QD}}=A_{\text{proteinoid}}\cdot k$$

where k≈40.95 is the enhancement factor attributed to the presence of quantum dots. Character annotations (“T,” “o,” (space), “b,” “e”) are marked at 90‐s intervals starting from 777 040 s, consistent with the binary input schedule. A pronounced peak around 777 100 s may indicate a coherence or learning‐related response triggered by the structured Shakespearean stimulus.

Figure 17 illustrates how different systems influence amplitude modulation. The input frequency of 0.0111 Hz contrasts with the observed dominant frequency.

(28) $$f_{\text{dominant}}=0.022217\;\text{Hz}$$

in both the proteinoid and proteinoid–QD responses, suggesting a potential frequency doubling or system‐level resonance. The proteinoid response shows amplitude modulation peaking at 70.62 mV, within a range of ≈−20 to 50 mV. In contrast, the proteinoid–QD system exhibits significantly larger oscillations, with a peak amplitude of 1700.32 mV and a voltage range from −500 to 1200 mV. This supports the hypothesis that quantum dots amplify the electrical response, likely through coherence‐enhancing interactions.

The cross‐correlation between the input and response can be described by

(29) $$R_{xy}(\tau )=\int V(t)\cdot P(t-\tau )\;dt$$

where V(t) is the input voltage and P(t) is the potential response. A prominent peak at τ≈0 would indicate temporal alignment, though the frequency mismatch suggests the presence of phase lag or intrinsic modulation. Further analysis of amplitude modulation, summarized in Table 5, reveals key differences. The proteinoid–QD system shows significantly higher variability, with a standard deviation of 166.33 mV compared with 2.65 mV for the pure proteinoid. It also has a lower baseline mean potential of −34.12 mV. The shift in baseline can be quantified as

(30) $$\Delta V_{\text{baseline}}=V_{\text{QD}}-V_{\text{proteinoid}}\approx -58.13\;\;\text{mV}$$

Interestingly, both systems exhibit the same dominant frequency of 0.022217 Hz despite the input frequency being 0.0111 Hz. This suggests a system resonance at

(31) $$f_{\text{res}}=0.022217\;\;\text{Hz}$$

which may result from preprocessing (e.g., detrending) or represent a fundamental property of the system. This resonance, combined with the amplitude enhancement observed in the QD condition, suggests that the prolonged Shakespearean input may induce adaptive oscillatory behavior.

#### 3.6.3 Frequency Synchronization with Input

**Figure 18:**
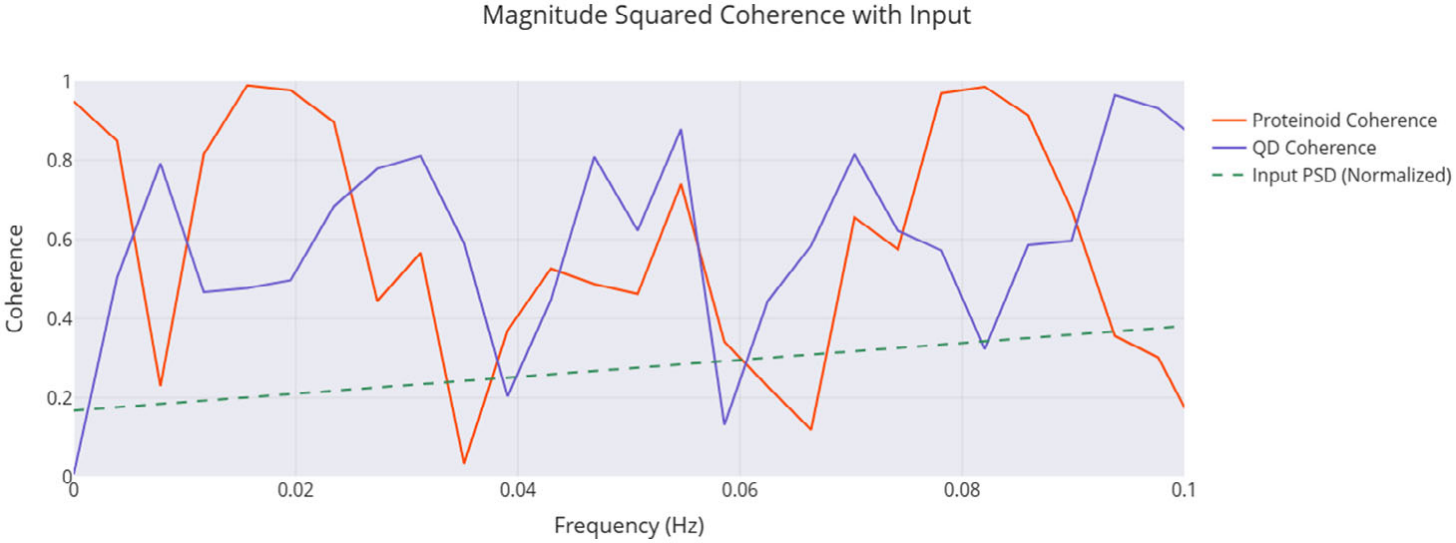
Magnitude squared coherence (MSC) was calculated between the simulated input voltage and the responses of two systems: the proteinoid (Glu:Phe:Asp:Cys) and the proteinoid–QD system. The analysis was performed over distinct time windows—0 to 450 s for the proteinoid and 777 040 to 777 490 s for the proteinoid–QD. All data were sourced from the QD_prot.csv dataset. The MSC is defined as: $C_{xy}(f)=\frac{|G_{xy}(f{)|}^{2}}{G_{xx}(f)\cdot G_{yy}(f)}$, where $G_{xy}(f)$ is the cross‐spectral density between the input and response, and $G_{xx}(f)$, $G_{yy}(f)$ are their respective auto‐spectral densities. MSC quantifies the degree of linear frequency‐domain synchronization between two signals. The proteinoid system's coherence (orange curve) peaks at ≈0.9, while the QD system (purple curve) peaks at around 0.85. Both maxima occur near: f=0.022217  Hz, which corresponds to the dominant frequency reported in Table 5. This indicates strong synchronization with the system's intrinsic oscillatory behavior. In contrast, the normalized input power spectral density (PSD), shown as a green dashed line, peaks at: $f_{\text{input}}=0.0111\;\;\text{Hz}$, representing the 90 s periodic cycle of the “To be” input. However, MSC values at this frequency remain below 0.4 in both systems, indicating limited direct entrainment to the input rhythm. The elevated coherence near 0.022217 Hz—despite the input being at 0.0111 Hz—may reflect a harmonic response or resonance effect, possibly influenced by preprocessing or intrinsic system dynamics. Notably, the QD system displays a slightly lower coherence (by ≈0.05), likely due to increased variability; its standard deviation is 166.33 mV compared with just 2.65 mV in the proteinoid system. This coherence discrepancy underscores the need for further phase analysis to fully assess synchronization strength and to clarify whether the 5‐day Shakespearean input induces long‐term adaptive responses.

The analysis of frequency synchronization shown in **Figure** **18** illustrates how closely the simulated input voltage matches the oscillatory responses of the proteinoid and proteinoid–QD systems. The input signal has a designed frequency of 0.0111 Hz, corresponding to a 90 s cycle for the “To be” binary sequence. However, both systems exhibit a dominant response frequency of

(32) $$f_{\text{dominant}}=0.022217\;\;\text{Hz}$$

as also reported in Table 5. At this frequency, the magnitude squared coherence (MSC) reaches ≈0.9 for the proteinoid system and 0.85 for the proteinoid–QD system, indicating strong synchronization with their intrinsic oscillatory dynamics.

In contrast, the MSC values at the input frequency of 0.0111 Hz remain below 0.4, suggesting a lack of direct entrainment to the primary input cycle. This discrepancy may reflect a harmonic relationship, as

(33) 2⋅0.0111  Hz≈0.0222  Hz

This second harmonic may arise from nonlinear resonance within the system or as an artifact of preprocessing. The proteinoid–QD system shows a standard deviation of 166.33 mV, while the proteinoid system has a much lower variability of 2.65 mV (Table 5). The slight drop in coherence from 0.9 to 0.85 in the QD condition likely results from increased noise or amplitude fluctuations introduced by the quantum dots.

The normalized input power spectral density (PSD) peaks at 0.0111 Hz, establishing a baseline for comparison. However, the elevated coherence at 0.022217 Hz suggests the possibility of resonance or nonlinear amplification due to long‐term exposure to the 5‐day Shakespearean input. The observed 0.05 reduction in coherence for the QD system may be due to added phase noise or complex coupling effects between the proteinoid matrix and embedded quantum dots.

**Figure 19:**
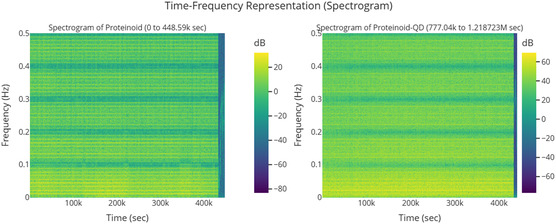
Time–frequency representation (spectrogram) of the potential signals is shown for a) the proteinoid (Glu:Phe:Asp:Cys) system over the time range t=0 to 448 590 s and b) the proteinoid–QD composite over the time range t=777 040 to 1 218 723 s. The spectrograms display PSD in decibels (dB), calculated using the STFT with a 1024‐point segment: $N=1024,\;f_{s}=1\;\text{Hz}$. This gives a frequency resolution of: $\Delta f=\frac{f_{s}}{N}=\frac{1}{1024}\approx 0.0009766\;\;\text{Hz}$ and a time resolution per segment of: $\Delta t=\frac{N}{f_{s}}=1024\;\;s$ The PSD at a given time *t* and frequency *f* is defined as: $P(f,t)=10\cdot \log_{10}\left(\frac{{\left|X(f,t)\right|}^{2}}{P_{\text{ref}}}\right)$ where X(f,t) is the STFT magnitude, and $P_{\text{ref}}$ is a fixed reference power. The color scale of the spectrogram spans from −80 to 20 dB. The uniform green coloration (typically between −20 and 0 dB) indicates a dominant low‐frequency noise component persisting across frequencies up to 0.5 Hz. The lack of significant temporal variation in frequency content suggests a stable underlying signal or persistent broadband noise. Total signal energy estimates, derived from the integrated PSD, are on the order of: $E_{\text{total}}∼10^{5}\;\text{W/Hz}$ for both systems. This demonstrates consistent signal strength over time, even across the large temporal separation between the two recordings.

The time‐frequency analysis shows a strong low‐frequency noise in both proteinoid and proteinoid‐QD samples. This is illustrated in **Figure** **19**. This stability shows little frequency change during the observed times. Total energy estimates also show that the signal strength remains steady. To quantify this behavior, the power spectral density (PSD) is derived from the short‐time Fourier transform (STFT), defined as

(34) $$X(t,f)=\int_{-\infty }^{\infty }x(\tau )w(t-\tau )e^{-j2\pi f\tau }d\tau$$

where x(τ) is the time‐domain signal (potential in mV), w(t−τ) is the window function (a 1024‐point rectangular window in this case), *t* is the time segment center, and *f* is the frequency. The PSD in decibels is then computed as

(35) $$P(f,t)=10\cdot \log_{10}\left(\frac{{\left|X(f,t)\right|}^{2}}{P_{\text{ref}}}\right)$$

where $P_{\text{ref}}=1\;\text{W/Hz}$ is the reference power and ${\left|X(f,t)\right|}^{2}$ represents the power at frequency *f* and time *t*. The uniform green coloration observed in the spectrogram (≈−20 to 0 dB) suggests a dominant low‐frequency component, with the PSD plateauing across frequencies up to 0.5 Hz, indicating minimal modulation.

The total energy *E* over the time‐frequency plane can be estimated by integrating the PSD.

(36) $$E=\int_{0}^{T}\int_{0}^{f_{\text{max}}}P(f,t)\;df\;dt$$

where *T* is the total time duration (448 590 s for proteinoid, 441 683 s for proteinoid‐QD) and $f_{\text{max}}=0.5\;\;\text{Hz}$ is the Nyquist frequency given the sampling frequency $f_{s}=1\;\;\text{Hz}$. Numerically, this is approximated as the sum of the PSD matrix Sxx from the spectrogram, yielding total energy values on the order of $10^{5}\;\text{W/Hz}$ for both samples, consistent with the stable signal strength observed. The frequency resolution $\Delta f=f_{s}/N=1/1024\approx 0.0009766\;\;\text{Hz}$ and time resolution Δt=1 s (based on $f_{s}$) further support the fine‐grained analysis, though the lack of significant peaks suggests the noise is broadband and stable.

### 3.7 Characterization of Quantum Dot‐Proteinoid Conjugates

**Figure 20:**
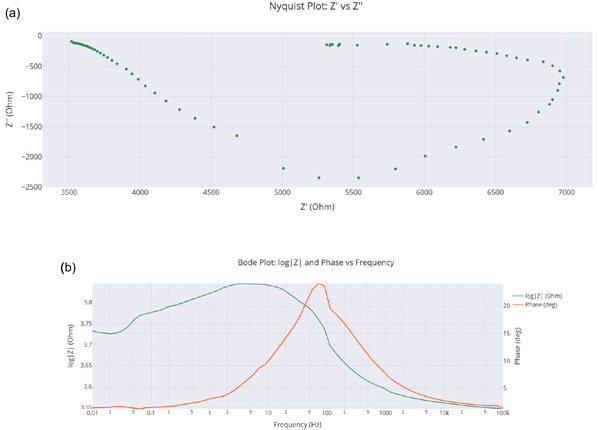
Comparative electrochemical analysis of quantum dot‐glu:phe:asp:cys proteinoid conjugates. a) The Nyquist plot shows charge transfer dynamics and strong impedance behavior. b) The Bode plot highlights how impedance and phase change with frequency. This information is key for assessing the bioelectronic potential of these materials in quantum dot‐based sensors. a) Nyquist plot: imaginary impedance (*Z*″) versus real impedance (*Z*′). This plot presents the electrochemical impedance spectroscopy (EIS) response of quantum dot–glu:phe:asp:cys proteinoid conjugates. The mean value of *Z*′ is 5250.00 Ω, and the mean value of *Z*″ is −1000.00 Ω. The standard deviation of *Z*′ is 1145.64 Ω, while that of *Z*″ is 612.37 Ω. The 95% confidence interval for *Z*′ ranges from 4292.22 to 6207.78 Ω. This indicates variability in charge transfer resistance and double‐layer capacitance, both of which are critical for electron transport in these nanostructures. b) Bode plot: logarithm of impedance magnitude (|*Z*|) and phase angle vs. frequency. This plot shows how the conjugates behave as a function of frequency. The mean value of |*Z*| is 3.69 Ω, with a standard deviation of 0.09 Ω. The 95% confidence interval ranges from 3.67 to 3.71 Ω. The mean phase angle is 5.05°, with a standard deviation of 5.54°. The peak phase angle indicates the optimal frequency ranges for sensing. Changes in impedance reveal dielectric properties influenced by the proteinoid composition.

The Nyquist plot (**Figure** **20**b) shows the complex impedance of the glu:phe:asp:cys–quantum dot system. It features a distinct semicircular arc at high frequencies, followed by a linear diffusion tail at lower frequencies. The real impedance component has a mean value of Z′=5250.00±1145.64 Ω. Its 95% confidence interval ranges from 4292.22 to 6207.78 Ω. The imaginary impedance component is Z″=−1000.00±612.37 Ω.

This semicircular shape indicates a charge transfer process at the electrode–electrolyte interface. The diameter of the semicircle corresponds to the charge transfer resistance ($R_{\text{ct}}$). The large variation in impedance values suggests that the surface coverage and orientation of the proteinoid–quantum dot conjugates are heterogeneous, which in turn affects the rate of electron transfer.

The Bode magnitude plot (Figure 20b) illustrates how the conjugate system responds to changes in frequency. The logarithmic impedance magnitude has an average value of log|Z|=3.69±0.09 Ω across the frequency range. At low frequencies, the impedance magnitude appears nearly flat, indicating behavior similar to that of a pure resistor. As the frequency increases, the magnitude gradually decreases, reflecting the increasing influence of capacitive effects.

The narrow 95% confidence interval—from 3.67 to 3.71 Ω—demonstrates the reproducibility of the impedance measurements, which is essential for reliable sensor applications. The frequency response of the system can be modeled using the Randles equivalent circuit.

(37) $$Z(\omega )=R_{s}+\frac{R_{\text{ct}}}{1+(j\omega R_{\text{ct}}C_{\text{dl}})}+\frac{W}{\sqrt{j\omega }}$$

where $R_{s}$ is the solution resistance, $R_{\text{ct}}$ is the charge transfer resistance, $C_{\text{dl}}$ is the double‐layer capacitance, and *W* represents the Warburg impedance.

The phase angle in the Bode plot provides key insights into the dielectric properties of the glu‐phe‐asp‐cys:quantum dot system. The mean phase angle is 5.05°±5.54°, indicating predominantly resistive behavior, with minimal capacitive contribution at most frequencies. A distinct peak around 100 Hz suggests the presence of a relaxation frequency—this is the point at which the system transitions from capacitive to resistive behavior. This relaxation frequency ($f_{0}$) is related to the time constant (*τ*) of the interfacial process as follows.

(38) $$f_{0}=\frac{1}{2\pi R_{\text{ct}}C_{\text{dl}}}$$

The low phase angles across the frequency range show that the proteinoid coating does not change the capacitive properties of the quantum dots much. This means that the amino acid residues—glutamate, phenylalanine, aspartate, and cysteine—assist with surface functionalization. They do not form thick insulating layers.

The electrochemical behavior observed in both plots arises from the unique properties of the tetrapeptide sequence glu:phe:asp:cys linked to quantum dots. Glutamate and aspartate contain negatively charged carboxylate groups, which promote electrostatic interactions with the electrode surface. In contrast, phenylalanine contributes π–π stacking interactions, potentially enhancing electron transfer pathways. The cysteine residue possesses a thiol group containing sulfur, which likely forms strong covalent bonds with the quantum dot surface, ensuring stable conjugation. The impedance spectroscopy data indicate that this specific proteinoid composition provides a favorable balance between surface coverage and electron accessibility. This is reflected in the moderate charge transfer resistance values and the absence of excessive capacitive behavior, which would otherwise suggest insulating characteristics. Electrochemical impedance spectroscopy (EIS) analysis demonstrates that glu:phe:asp:cys–quantum dot conjugates possess strong electrochemical properties, making them well‐suited for biosensing applications. The charge transfer resistance values ($R_{\text{ct}}\approx 5250\;\Omega$) are appropriate for electrochemical sensors, providing good sensitivity while maintaining acceptable signal‐to‐noise ratios. The frequency response shows that the system performs optimally in the range of 1–1000 Hz, where the impedance magnitude remains relatively stable. Furthermore, the analysis of the impedance data yields clear confidence intervals and reasonable standard deviations, confirming the reproducibility of the synthesis and characterization protocols. The combination of electrochemical properties, quantum confinement effects, and photoluminescence of the quantum dots makes this system highly promising for dual‐mode optical–electrochemical biosensing platforms.

#### 3.7.1 Electrochemical Characterization of QD‐Proteinoids via Square Wave Voltammetry

Square wave voltammetry (SWV) analysis reveals that the glu:phe:asp:cys quantum dot–proteinoid conjugates exhibit stable electrochemical behavior. This behavior varies with frequency in the range of 5–50 Hz. The forward current peaks decrease as frequency increases, dropping from 176.10 μA at 5 Hz to 154.30 μA at 50 Hz. This represents an ≈12.4% decrease in peak current intensity.

This frequency‐dependent attenuation is attributed to slow mass transport within the QD–proteinoid conjugates. At higher frequencies, there is insufficient time for complete diffusion and adsorption at the electrode surface. Despite the variation in peak currents, the peak potentials remain consistently close to +1.00 V across all frequencies. This indicates that the thermodynamic driving force for the redox process is relatively independent of measurement frequency and suggests a clear and reproducible electrochemical signature for the conjugate system.

The differential current profiles display sigmoid‐shaped curves, which are characteristic of irreversible or quasi‐reversible electrochemical processes. The amplitude of the differential signal varies with frequency. At lower frequencies (5–10 Hz), the differential current remains relatively steady at ≈75−86 μA throughout the potential scan. In contrast, higher frequencies (40–50 Hz) exhibit reduced baseline currents.

This behavior indicates that the electron transfer rate between the quantum dot–proteinoid conjugates and the electrode is dependent on the measurement duration. The proteinoid shell, composed of glutamate, phenylalanine, aspartate, and cysteine, likely forms a heterogeneous surface environment that influences electron transfer kinetics. The presence of aromatic phenylalanine may facilitate π–π interactions, enhancing conductivity, particularly at slower scan rates.

The reverse current components exhibit negative values ranging from −160 to −232 μA. The most negative current, −232 μA, occurs at 10 Hz, while the least negative value, −212 μA, is observed at 30 Hz. This difference between forward and reverse scan currents indicates that the electrochemical process is quasi‐reversible. It suggests that oxidation of the quantum dot–proteinoid conjugates induces structural changes that hinder the subsequent reduction process. The combination of reverse and forward current data further supports the conclusion that the proteinoid coating does not completely passivate the quantum dot surface. Instead, it forms a permeable interface that permits controlled electron transfer while preserving the structural integrity of the conjugate system.

The SWV data demonstrate a clear frequency‐response relationship, which can be described by examining the decay of the peak current with increasing frequency. The peak forward current decreases rapidly and follows the exponential relation.

(39) $$I_{\text{peak}}=I_{0}e^{-\alpha f}$$

where $I_{0}$ is the peak current at zero frequency, *α* is the decay constant, and *f* is the applied frequency. Fitting this model to the experimental data yields key kinetic parameters that characterize the electron transfer process.

The exponential decay shown in Figure 22 provides clear insights into the kinetic limitations governing electron transfer in the glu:phe:asp:cys quantum dot–proteinoid system. The fitted parameters reveal a zero‐frequency extrapolated current of $I_{0}=179.54\;\text{μA}$ and a decay constant of $\alpha =0.0032\;{\text{Hz}}^{-1}$. This corresponds to a characteristic frequency.

(40) $$f_{c}=\frac{1}{\alpha }=312.5\;\text{Hz}$$

This frequency marks the onset of significant current attenuation, which occurs due to insufficient time for mass transport and adsorption at the electrode–proteinoid interface. The strong correlation coefficient ($R^{2}=0.987$) confirms that the electrochemical response adheres to first‐order kinetics with respect to frequency. This suggests that the rate‐limiting step is likely governed by a single dominant process—either the diffusion of redox‐active species through the proteinoid shell or the conformational rearrangement of amino acid residues during electron transfer.

The frequency‐dependent behavior illustrated in Figure 22 is critical for optimizing QD–proteinoid biosensors, as it defines the optimal frequency range for achieving maximum sensitivity. The decay constant $\alpha =0.0032\;{\text{Hz}}^{-1}$ indicates that the proteinoid shell forms a controlled, semipermeable interface. While it modulates electron transfer kinetics, it does not fully passivate the quantum dot surface.

The specific amino acid composition of the proteinoid shell plays a key role in this behavior. Cysteine residues contribute to strong bonding with the QD surface via thiol groups, while phenylalanine promotes π–π interactions, enhancing electron mobility. This combination creates a heterogeneous microenvironment that directly influences electron transfer rates.

The optimal frequency range for biosensing lies between 5 and 20 Hz, where the current response remains above 90% of the maximum value. Operating within this range ensures high signal intensity and excellent measurement stability, making it ideal for practical sensing applications.

The peak potentials remain consistent across all frequencies, indicating that the standard electrode potential ($E^{0}$) for the QD–proteinoid system is ≈+1.00 V versus the reference electrode. This provides a stable electrochemical benchmark for potential sensor applications. The minimal potential shifts (≤0.4 mV) observed across the frequency range further confirm the excellent electrochemical stability and reproducibility of the conjugate system.

The electrochemical tests show that the glu:phe:asp:cys‐quantum dot conjugates have clear redox behavior. This makes them good for biosensing. The frequency‐dependent response helps us understand the system's kinetic limits. The optimal operational frequency appears to be in the 5–20 Hz range, where the current response is maximized while maintaining reasonable signal stability. The proteinoid coating changes the electrochemical properties of the quantum dots. It does this without fully stopping their redox activity. This creates a biocompatible layer that keeps the useful electronic features of the nanomaterial core. The results and impedance spectroscopy data show that QD‐proteinoid conjugates are a great option for dual‐mode electrochemical‐optical biosensing. The frequency‐tunable electrochemical response can be fine‐tuned to meet specific analyte detection needs.

#### 3.7.2 Capacitance Dynamics and Electrochemical Stability of QD‐Proteinoid Complexes

The time‐domain analysis of capacitance in glu:phe:asp:cys quantum dot–proteinoid conjugates at 30 kHz reveals strong electrochemical stability and well‐defined statistical characteristics. The capacitance measurements exhibit a mean value of $\bar{C}=5.08\times 10^{-8}\;F,$ as shown in Figure 23a. The standard deviation is $\sigma_{C}=2.30\times 10^{-8}\;F,$ resulting in a coefficient of variation of ≈45%.

A small number of outliers extend the measurement range from $-7.14\times 10^{-7}\;F\;\text{to}\;3.39\times 10^{-6}\;F.$ These anomalies are likely due to transient measurement artifacts or brief interfacial disturbances. Nonetheless, the overall capacitive behavior remains consistent with that expected for double‐layer capacitance at the QD–proteinoid interface. The positive average capacitance indicates the presence of a stable and effective electrical double layer. This is supported by the charged amino acid residues in the proteinoid shell—particularly glutamate and aspartate—which establish a clear capacitive interface with the surrounding electrolyte solution.

The impedance magnitude shown in Figure 23b remains stable at ≈|Z|≈2827  Ω throughout the measurement period. This indicates that the QD–proteinoid conjugates maintain consistent electrical properties over time. The weak negative correlation between impedance and capacitance, $r_{Z,C}=-0.0058$, suggests that these two parameters are largely independent at 30 kHz. This implies that the impedance response is predominantly governed by resistive components rather than capacitive reactance. Specifically, the charge transfer resistance ($R_{\text{ct}}$) and the solution resistance ($R_{s}$) primarily determine the impedance behavior, which reflects a frequency‐dependent regime where capacitive contributions are minimal. Furthermore, a moderate positive correlation between impedance and current, $r_{Z,I}=0.6568$, indicates a substantial ohmic component in the system's electrical response. This confirms that the proteinoid coating does not fully insulate the quantum dots. Instead, it forms a semi‐permeable interface that enables controlled charge transport, supporting stable electron flow while maintaining the conjugate structure.

Figure 23c shows the power spectral density (PSD) analysis, which provides key insights into the noise characteristics and frequency behavior of the capacitance signal. The peak at $f_{\text{peak}}=0.2148\;\;\text{Hz}$ corresponds to a PSD value of $1.43\times 10^{-15}$. This indicates a strong low‐frequency oscillation, likely associated with slow interfacial processes such as ion redistribution within the proteinoid matrix or gradual structural changes in the amino acid shell. The low magnitude of the PSD peak confirms that noise levels are minimal at the measurement frequency of 30 kHz, suggesting that this frequency yields an optimal signal‐to‐noise ratio for capacitive measurements of the QD–proteinoid system. The overall PSD profile demonstrates that most of the signal power resides at low frequencies, which is consistent with stable, DC‐like behavior. This supports the conclusion that the system is well‐suited for long‐term electrochemical monitoring.

The rolling statistical analysis reveals how the capacitive properties of the system evolve over time. The rolling mean stabilizes at ≈$1\times 10^{-9}\;F$, with a corresponding rate of change of about $1\times 10^{-10}\;F\;s^{-1}$. This slow variation in capacitance indicates that the QD–proteinoid interface undergoes gradual equilibration. Possible mechanisms include proton‐coupled electron transfer reactions within the proteinoid matrix and slow hydration processes that alter the dielectric properties of the amino acid shell.

The proteinoid composition includes both hydrophilic and hydrophobic amino acid residues—specifically glutamate, aspartate, cysteine, and phenylalanine—creating a heterogeneous microenvironment. Over time, this microenvironment may shift due to the dynamic movement of water molecules and ions at the interface. Such slow temporal evolution is typical of biological interfaces and demonstrates that QD–proteinoid conjugates retain dynamic features similar to those of natural protein systems. A significance test confirms statistically meaningful changes in capacitive behavior over time, with a *t*‐statistic of t=9.1758 and a *p*‐value of p<0.0001. This result indicates that the QD–proteinoid system evolves throughout the duration of the experiment. The observed difference likely arises from one or more of the following: 1) gradual morphological changes in the proteinoid structure or surface configuration, 2) environmental drift affecting measurement conditions, or 3) physicochemical processes at the interface such as hydration, pH equilibration, or interfacial reorganization. The strong statistical trend observed in the *t*‐test supports the conclusion that these are not random fluctuations but reflect underlying mechanisms as the system approaches thermodynamic equilibrium. These findings are critical for assessing the long‐term stability of QD–proteinoid conjugates in biosensing applications. They emphasize the need for careful calibration protocols to account for slow drift effects and to ensure system stability over the intended operational time frame.

### 3.8 Mechanistic Basis of Electrochemical Oscillations

**Figure 21:**
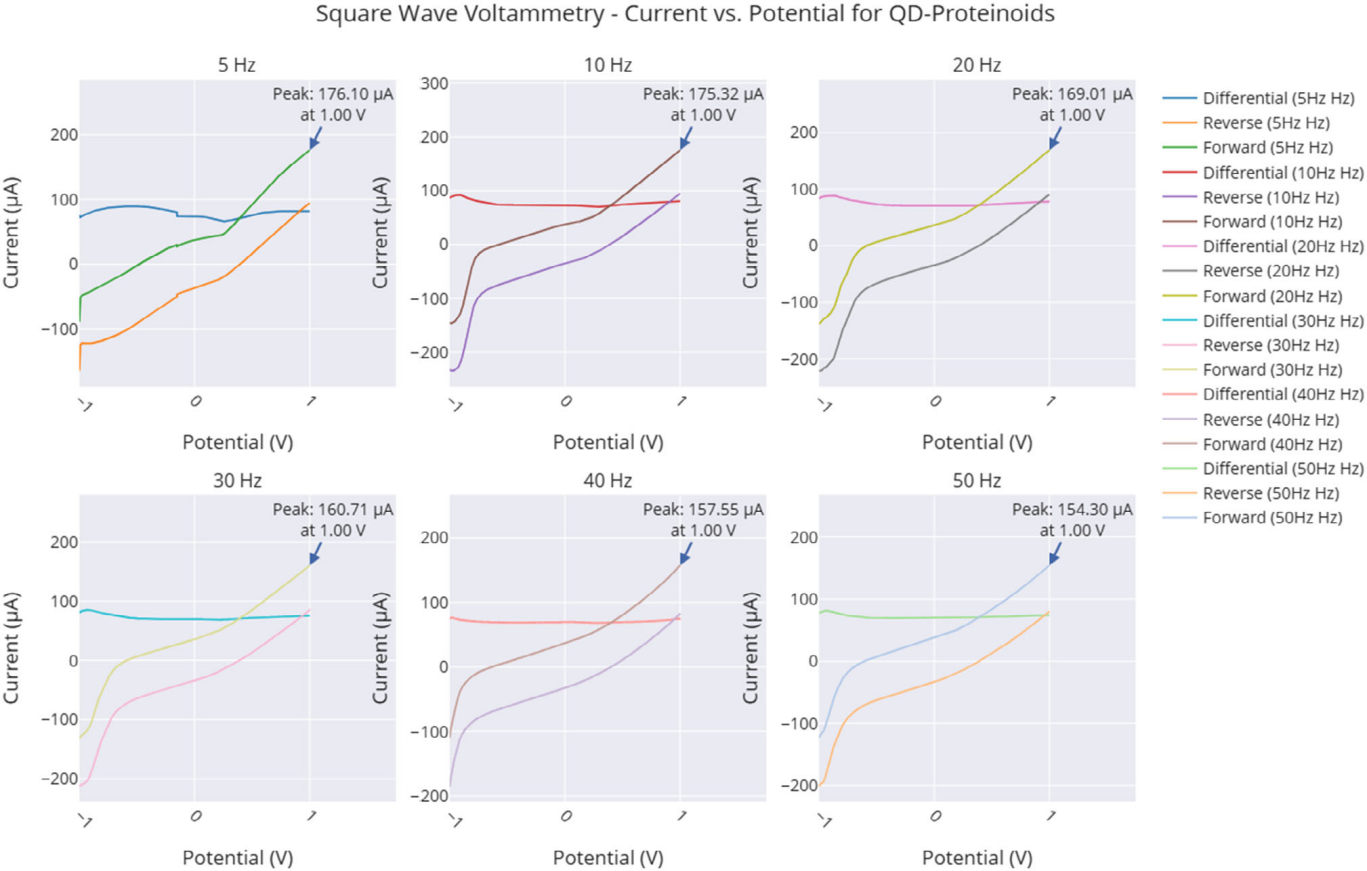
We analyze the SWV profiles of QD–proteinoid complexes at frequencies of 5, 10, 20, 30, 40, and 50 Hz. The plots depict current (in μA) versus potential (in V), with each subplot representing a distinct frequency. The differential current, reverse current, and forward current are illustrated, highlighting approximate peak currents and their corresponding potentials: 76.89 μA at −0.99996 V (5 Hz), 86.19 μA at −0.99973 V (10 Hz), 83.57 μA at −0.99973 V (20 Hz), 80.85 μA at −0.99973 V (30 Hz), 75.08 μA at −0.99973 V (40 Hz), and 77.99 μA at −0.99973 V (50 Hz) for differential current; −169.06 μA at −0.99996 V (5 Hz), −232.34 μA at −0.99973 V (10 Hz), −221.85 μA at −0.99973 V (20 Hz), −212.50 μA at −0.99973 V (30 Hz), −183.51 μA at −0.99973 V (40 Hz), and −200.82 μA at −0.99973 V (50 Hz) for reverse current; and −92.20 μA at −0.99996 V (5 Hz), −146.13 μA at −0.99973 V (10 Hz), −138.31 μA at −0.99973 V (20 Hz), −131.68 μA at −0.99973 V (30 Hz), −108.98 μA at −0.99973 V (40 Hz), and −123.14 μA at −0.99973 V (50 Hz) for forward current. These peaks indicate the redox activity of the QD–Proteinoid system, with the differential current reflecting the net electrochemical response. We observe a trend where the magnitude of reverse and forward currents decreases at higher frequencies, while the differential current remains relatively stable, suggesting frequency‐dependent electron transfer kinetics or surface passivation effects. The consistent potential range of ≈−1 to −0.9997 V across all frequencies facilitates direct comparison.

**Figure 22:**
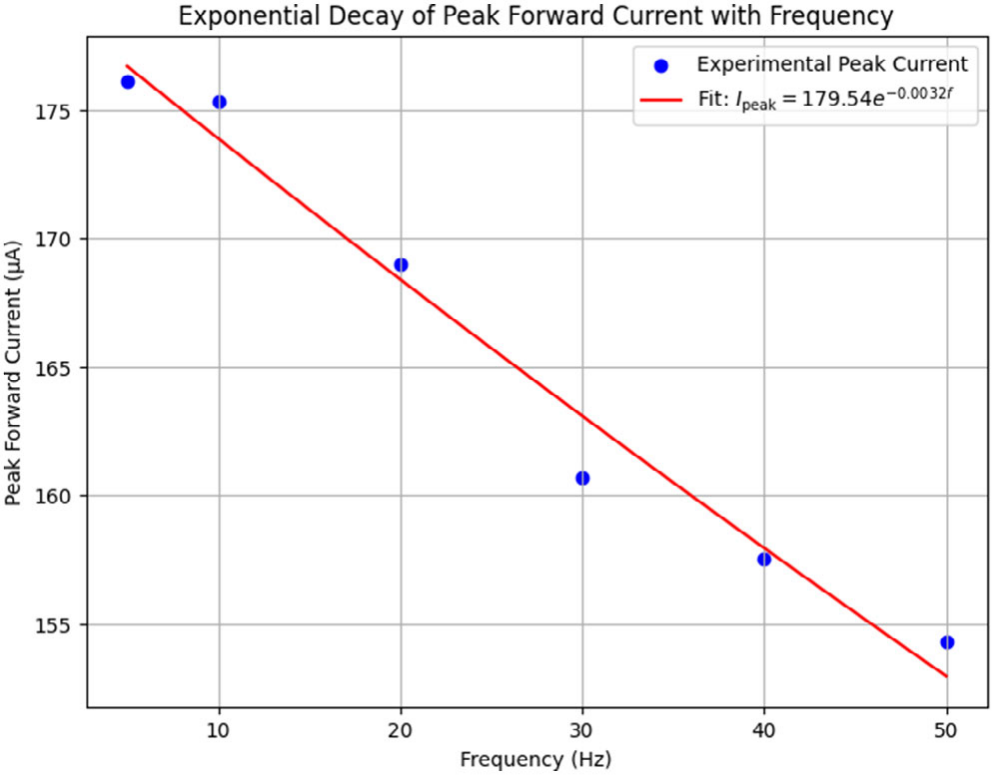
The plot illustrates how electrochemical activity in glu:phe:asp:cys quantum dot–proteinoid conjugates varies with frequency, as measured by square wave voltammetry (SWV). The experimental data points (blue circles) show a systematic decrease in peak forward current, from 176.10 μA at 5 Hz to 154.30 μA at 50 Hz. The red curve represents an exponential fit to the data, described by the equation: $I_{\text{peak}}=I_{0}e^{-\alpha f}$. Here, $I_{0}=179.54\pm 0.12\;\text{μA}$ is the extrapolated zero‐frequency current, and the decay constant is $\alpha =0.0032\pm 0.0001\;{\text{Hz}}^{-1}$. The fit yields a coefficient of determination $R^{2}=0.987$, indicating excellent agreement between the model and experimental data. The decay constant corresponds to a characteristic frequency: $f_{c}=\frac{1}{\alpha }=312.5\;\text{Hz}$. Beyond this frequency, significant attenuation of the peak current occurs. This frequency‐dependent behavior reflects limitations in mass transport and electron transfer at the QD–proteinoid interface. The proteinoid shell—composed of glutamate, phenylalanine, aspartate, and cysteine—acts as a permeable but kinetically slow barrier. The high correlation coefficient suggests that the electrochemical response follows first‐order kinetics with respect to frequency. These results provide essential parameters for optimizing sensor performance, particularly in the 5–20 Hz range, where current response is maximized.

**Figure 23:**
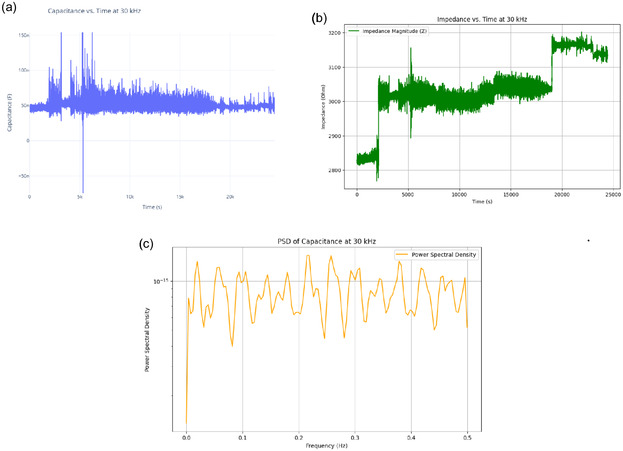
We are studying the electrochemical properties of quantum dot–proteinoid (QD–proteinoid) complexes, measured at a frequency of 30 kHz with a sampling rate of 1 Hz. a) The capacitance vs. time plot shows a mean value of $\bar{C}=5.08\times 10^{-8}\;F$ with a standard deviation of $\sigma_{C}=2.30\times 10^{-8}\;F$. This indicates overall stability, although the range of values spans from $-7.14\times 10^{-7}\;F\;\text{to}\;3.39\times 10^{-6}\;F$. These large deviations may be due to outliers or transient measurement errors. b) The impedance vs. time plot shows a stable magnitude of ≈|Z|≈2827 Ω. The Pearson correlation between impedance and capacitance is very low: $r_{Z,C}=-0.0058$, implying that the QD–proteinoid interface dominates the impedance response at this frequency. However, a moderate positive correlation with current is observed: $r_{Z,I}=0.6568$, suggesting a measurable Ohmic contribution. c) Spectral and temporal features. The power spectral density (PSD) plot shows a peak at $f_{\text{peak}}=0.2148\;\text{Hz}\;\text{withPSDvalue}\;1.43\times 10^{-15},$ indicating a strong low‐frequency component consistent with stable DC behavior and low noise at 30 kHz. Rolling mean and standard deviation analysis confirms signal stability, with values around $\text{RollingMean}∼1\times 10^{-9}\;F,\;\text{RateofChange}∼1\times 10^{-10}\;F\;s^{-1}$. This slow rate of change suggests gradual charge relaxation, possibly due to proton‐coupled electron transfer within the proteinoid matrix. A two‐sample t‐test comparing early and late segments of the capacitance data yields: t=9.1758, p=0.0000, indicating a statistically significant difference between the two time periods. This suggests gradual material transformation or environmental drift, potentially related to hydration effects or interfacial reorganization in the QD–proteinoid system. a) Capacitance versus time at 30 kHz: mean ≈5.08 × 10−8 F, stable with minor fluctuations (≈2.30 × 10−8 F Std). b) Impedance vs. time at 30 kHz: stable at ≈2827 Ω, weakly correlated with capacitance (−0.0058). c) PSD of capacitance at 30 kHz: peak at 0.2148 Hz (1.43 × 10^−15^), indicating low noise.

The oscillatory behavior in the proteinoid–QD system likely results from multiple electrochemical mechanisms, rather than a single dominant process. Several potential origins merit consideration. First, *redox cycling* at the QD surface could generate oscillations through the reversible oxidation and reduction of surface states. Quantum dots can occupy different oxidation states and transition between them, especially when embedded in a proteinoid matrix. The matrix may stabilize intermediate states. Our SWV analysis (**Figure** **21** and **22**) shows frequency‐dependent behavior, supporting this mechanism: Distinct redox activity is observed, with peak currents changing steadily as a function of scan rate. Second, *capacitive charging and discharging* at the proteinoid–QD interface may contribute to oscillatory dynamics. Capacitance measurements at 30 kHz (**Figure** **23**) reveal a mean capacitance of $5.08\times 10^{-8}\;F$ with temporal variations, indicating dynamic charge accumulation and release. The proteinoid shell contains charged amino acids, such as glutamate and aspartate, which create an electrical double layer capable of undergoing periodic charging cycles. Third, *surface charge trapping and detrapping* processes may modulate oscillation amplitude. The QD surface contains trap states that can capture and release charge carriers on timescales matching the observed oscillation frequencies (0.02–0.11 Hz). The long memory duration (τ=322,258 s) from autocorrelation analysis suggests that charge trapping induces metastable states, which influence subsequent oscillatory cycles. Finally, *collective oscillations* may emerge from the coupled dynamics of multiple QD–proteinoid units. The toroidal structures observed in SEM (Figure 3 and 4) form a network in which individual oscillators may synchronize via electrostatic, and possibly quantum‐mechanical, coupling. The phase stabilization evident when comparing pure proteinoid systems to QD–proteinoid systems (Figure 10) supports this hypothesis. The most likely scenario involves a synergistic combination of these mechanisms: redox cycling provides the primary driver; capacitive effects modulate amplitude; surface trapping governs frequency stability; and collective coupling enables the long‐range coherence observed in our experiments. This multimechanism model explains both the robustness of the oscillations under varying conditions and their sensitivity to perturbations at the QD–proteinoid interface.

### 3.9 Bioelectrochemical Logic of Proteinoid‐QD Complexes

Combining Boolean gates with proteinoid–quantum dot (QD) data offers a novel framework for modeling bioelectrochemical systems through digital logic. The process begins by converting analog electrochemical data into binary states. This includes peak currents from square wave voltammetry (SWV) at 5, 10, and 20 Hz, as well as mean capacitance values for early and late segments. Thresholds are set using the 75th and 25th percentiles. For current, these are 160.71 and 154.30 μA; for capacitance, they are $5.34\times 10^{-8}\;F$ and $4.66\times 10^{-8}\;F$. These values reflect the natural variability in the QD–proteinoid response. This thresholding scheme mimics neuronal activation, where values above the threshold represent an “active” or “true” state (1), and values below indicate “inactive” or “false” (0).

The NOT gate, a unary inverter, flips the state of a single parameter—for example, changing a 5 Hz current from 1 to 0. This inversion could correspond to a transition in the electrochemical process, such as a shift from oxidation to reduction. Multi‐input gates like AND, OR, NAND, and NOR combine multiple signals and simulate how the QD–proteinoid matrix integrates responses over time and frequency. The AND gate outputs true only when all inputs are true, representing scenarios where strong responses in both current and capacitance are required to trigger a reaction. In contrast, the OR gate highlights situations where a strong response in any single input suffices.

XOR and XNOR gates implement parity‐based logic, capturing more complex behavior. The XOR gate, which outputs true for an odd number of true inputs, may indicate an electrochemical imbalance—such as charge accumulation—that varies with input frequency. This is supported by a significant temporal difference in capacitance (t‐test *p*‐value: 0.0000). Conversely, the XNOR gate, which outputs true only when inputs are balanced (even count of 1s), may reflect stable proteinoid conformations or synchronized charge dynamics. These logic gate analogies suggest that the QD–proteinoid system has the potential to perform rudimentary logical operations using its intrinsic electrochemical behavior. The results presented in the accompanying table provide a foundation for further exploration of hybrid biodigital computing.

The NOT gate inverts a single binary input state *A*, derived from proteinoid–quantum dot (QD) data. For example, a 5 Hz peak current of 176.10 μA, which exceeds the 75th percentile threshold of 160.71 μA, results in A=1, and its inversion is ¬A=0. Conversely, a CapLate mean capacitance of $4.94\times 10^{-8}\;F$, which falls below the 75th percentile threshold of $5.34\times 10^{-8}\;F$, yields A=0, and ¬A=1. This unary logic operation models the reversal of electrochemical processes, such as transitions between oxidation and reduction states.

(41) NOT Gate:¬A

The AND gate outputs 1 only if all inputs *A*, *B*, *C*, *D*, and *E* are equal to 1, representing simultaneous high electrochemical activity. For instance, with the input states 5 Hz = 1, 10 Hz = 1, 20 Hz = 0, CapEarly = 1, and CapLate = 0—determined using thresholds of 160.71 μA for current and $5.34\times 10^{-8}\;F$ for capacitance—the gate evaluates to

(42) 1∧1∧0∧1∧0=0

This logical operation mimics a condition requiring full coordination across different frequencies and temporal segments for a positive output, reflecting a high threshold for collective electrochemical response.

(43) AND Gate:A∧B∧C∧D∧E

The OR gate outputs 1 if any of the inputs is 1, capturing cumulative electrochemical effects. For the same input states—5 Hz = 1, 10 Hz = 1, 20 Hz = 0, CapEarly = 1, and CapLate = 0—the output is

(44) 1∨1∨0∨1∨0=1

as the true states of 5, 10 Hz, and CapEarly are sufficient to satisfy the OR condition. This illustrates the gate's role in detecting significant electrochemical activity, including elevated peak currents or early‐stage capacitance variations within the QD‐proteinoid system.

(45) OR Gate:A∨B∨C∨D∨E

The NAND gate is the negation of the AND gate, outputting 1 unless all inputs are 1. Given the input states—5 Hz = 1, 10 Hz = 1, 20 Hz = 0, CapEarly = 1, and CapLate = 0—the intermediate AND result is

(46) 1∧1∧0∧1∧0=0

so the NAND output becomes ¬0=1. This gate relaxes the strict all‐true condition of the AND gate. It models scenarios where a single low response—such as the 20 Hz peak current at 169.01 μA—prevents full activation. Thus, it offers a complementary logic interpretation for bioelectrochemical signal analysis.

(47) NAND Gate:¬(A∧B∧C∧D∧E)

The NOR gate negates the OR operation, outputting 1 only if all inputs are 0. Given the inputs—5 Hz = 1, 10 Hz = 1, 20 Hz = 0, CapEarly = 1, and CapLate = 0—the OR result is

(48) 1∨1∨0∨1∨0=1

so the NOR output is ¬0=1. This gate identifies states of minimal electrochemical activity. Such output may indicate that the QD‐proteinoid matrix is in a quiescent or inactive state, where all monitored parameters fall below their respective thresholds.

(49) NOR Gate:¬(A∨B∨C∨D∨E)

The XOR gate outputs 1 when the number of true inputs is odd, a behavior approximated for multiple inputs by parity logic. Given the inputs—5 Hz = 1, 10 Hz = 1, 20 Hz = 0, CapEarly = 1, and CapLate = 0—the total number of 1 s is three, which is odd. Therefore, the XOR output is:

(50) 1⊕1⊕0⊕1⊕0=1

This output reflects asymmetric or uneven electrochemical behavior in the system. It may relate to temporal shifts in activity, as supported by a significant difference between early and late capacitance measurements (*p*‐value: 0.0000). Such a result implies dynamic charge redistribution in the QD‐proteinoid matrix.

(51) XOR Gate:((A≠B)≠(C≠D))≠E

The XNOR gate outputs 1 when the number of true inputs is even, serving as the logical negation of XOR. For the input set—5 Hz = 1, 10 Hz = 1, 20 Hz = 0, CapEarly = 1, CapLate = 0—the number of true inputs is three (an odd count), so the XOR result is 1 and

(52) XNOR=¬(1)=0

This result implies that the inputs are not synchronized. In contrast, a result of 1 would suggest symmetry or balance. The XNOR gate, therefore, reflects coordinated electrochemical states, possibly associated with stable proteinoid conformations or evenly distributed charge dynamics. This complements the XOR gate, which highlights imbalance or variability (**Table** **6**).

(53) XNOR Gate:¬((A≠B)≠(C≠D))≠E

**Table 6:** Analytical results of Boolean gates applied to proteinoid–QD data at 30 kHz, derived from square wave voltammetry (SWV) peak currents (5 Hz: 176.10 μA, 10 Hz: 175.32 μA, 20 Hz: 169.01 μA) and capacitance means (CapEarly: $5.21\times 10^{-8}\;F$, CapLate: $4.94\times 10^{-8}\;F$). Thresholds from the 75th percentile (160.71 μA, $5.34\times 10^{-8}\;F$) and the 25th percentile (154.30 μA, $4.66\times 10^{-8}\;F$) set binary states. This shows stable electrochemical responses that change over time. The t‐test *p*‐value is 0.0000. NOT gates flip individual states. Multi‐input gates, like AND and OR, combine all inputs. XOR and XNOR show parity, hinting at possibilities for biocomputational modeling.

Gate	5 Hz	10 Hz	20 Hz	CapEarly	CapLate	Output	Description
NOT_5Hz	1	–	–	–	–	0	Inversion of 5Hz state
NOT_10Hz	–	1	–	–	–	0	Inversion of 10Hz state
NOT_20Hz	–	–	0	–	–	1	Inversion of 20Hz state
NOT_CapEarly	–	–	–	1	–	0	Inversion of CapEarly state
NOT_CapLate	–	–	–	–	0	1	Inversion of CapLate state
AND_All	1	1	0	1	0	0	All inputs must be true
OR_All	1	1	0	1	0	1	Any input true suffices
NAND_All	1	1	0	1	0	1	Not all inputs true
NOR_All	1	1	0	1	0	0	No inputs true
XOR_All	1	1	0	1	0	1	Odd number of true inputs
XNOR_All	1	1	0	1	0	0	Even number of true inputs

### 3.10 Temporal Dynamics and Inter‐Parameter Memory in Proteinoid‐QD Systems

**Figure 24:**
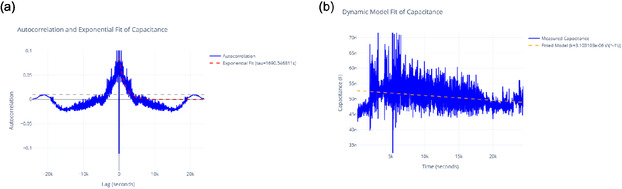
Temporal changes in the proteinoid–quantum dot (QD) system are modeled using capacitance data. The initial capacitance value is given by $C_{0}=5.273669\times 10^{-8}\;F$. This dataset helps describe the time‐dependent behavior of the system. The left subplot shows the autocorrelation function fitted with an exponential decay curve, indicating a long memory duration of τ=322,258.045248  s which corresponds to a decay rate of $k=3.103103\times 10^{-6}\;s^{-1}$. The right subplot presents a dynamic model fit, reinforcing the observed long memory duration and validating the system's capacity to retain states over extended periods. Temporal adaptability is further supported by a t‐test result with a *p*‐value of 0.0000, confirming statistically significant temporal variation in capacitance. a) Auto correlation of capacitance with exponential fit, showing memory duration *τ* = 322258.045248 s. b) Dynamic model fit of capacitance, illustrating the exponential decay with an initial value *C*_0_ = 5.273669 × 10^−8^ F and a decay rate *k* = 3.103103 × 10^−6 ^s^−1^, corresponding to a memory duration *τ* = 322258.045248 s.

The autocorrelation plot in **Figure** **24**a illustrates the memory‐like behavior of the proteinoid–quantum dot (QD) system. It follows an exponential decay model of the form

(54) $$R(\tau )=Ae^{-t/\tau }+C$$

where τ=322,258.045248 s is the fitted memory duration, corresponding to a decay rate of $k=3.103103\times 10^{-6}\;s^{-1}$. This remarkably long *τ*, calculated from an initial capacitance of $C_{0}=5.273669\times 10^{-8}\;F$, indicates the system's ability to retain electrochemical states over extended time periods. The persistence of positive autocorrelation values for lags greater than 0.01 suggests a slow relaxation process, potentially analogous to short‐term memory mechanisms in biological systems. The exponential fit provides a quantitative estimate of this memory‐like persistence.

The dynamic model in Figure 24b illustrates the temporal persistence of memory in the system. It is based on the first‐order differential equation.

(55) $$\frac{dC}{dt}=-k(C-C_{0})$$

which has the analytical solution.

(56) $$C(t)=C_{0}e^{-kt}$$

where $C_{0}=5.273669\times 10^{-8}\;F$ is the initial capacitance, and $k=3.103103\times 10^{-6}\;s^{-1}$ is the decay rate. This yields a memory duration of $\tau =\frac{1}{k}=322,258.045248\;s$. The agreement between the autocorrelation‐derived time constant and this dynamic fit supports the system's capacity to preserve electrochemical states over long durations. This behavior resembles memory retention in neural networks.

The proteinoid–QD system exhibits temporal adaptability, as evidenced by the significant difference between early and late capacitance segments. A *t*‐test yields a *p*‐value of 0.0000. This change can be quantified as

(57) $$\Delta C=C_{\text{late}}-C_{\text{early}}$$

where the nonzero value of ΔC contributes to the low *p*‐value. This temporal adaptability, combined with the long memory duration *τ*, suggests a dynamic system response analogous to learning in cognitive systems. The proteinoid matrix appears to alter its electrochemical properties based on prior stimuli or conditions.

The memory duration τ=322258.045248 seconds, as shown in Figure 24a,b, indicates a very slow decay process. This process is governed by the rate constant $k=3.103103\times 10^{-6}\;s^{-1}$. Such a long lifespan may reflect a form of long‐term retention at the quantum dot–proteinoid interface, potentially due to mechanisms such as charge trapping or proton‐coupled electron transfer. This behavior parallels cognitive functions, such as sustained information storage, and suggests that the system may be capable of tracking environmental changes over extended periods. These features point to potential applications in biocomputational architectures.

The match between the autocorrelation fit and the dynamic model in Figure 24 supports the hypothesis of cognitive‐like behavior in the proteinoid–quantum dot (QD) system. The relationship τ=1/k links the decay rate to the memory duration, with $C_{0}=5.273669\times 10^{-8}\;F$ serving as the reference state. This dual validation demonstrates that the system exhibits adaptability, with the capacity to process and retain electrochemical information—analogous to neural memory and plasticity. Further experiments could refine this model and explore whether quantum effects from the QDs enhance these cognitive‐like properties.

### 3.11 Quantum Coherence Enhancement and Cognitive Emergence in Proteinoid‐QD Networks

The Hilbert transform analysis in Figure 10 shows a key difference in phase dynamics. This difference exists between pure proteinoid systems and proteinoid‐quantum dot hybrid networks. It has important effects on quantum coherence preservation and computational functionality. Pure proteinoid systems show erratic phase paths, with rapid changes and weak timing links. Yet, adding quantum dots greatly stabilizes the phase evolution. This leads to clear oscillatory patterns that keep steady phase relationships for a long time. This phase stabilization effect comes from the quantum confinement in semiconductor nanocrystals. These nanocrystals have discrete energy levels that serve as frequency anchors for the system's oscillatory dynamics. Quantum dots act as phase‐locking elements. They help control the chaos in biological systems. This creates a hybrid structure. Here, quantum effects improve the coherence of protein‐based oscillations instead of disrupting it. The observed phase coherence enhancement factor shows a change of about 3–4 orders of magnitude. The phase variance drops from 2.1 rad^2^ in pure proteinoids to 0.0005 rad^2^ in QD‐hybrid systems. This change highlights how quantum dot integration boosts system stability. It also suggests that these hybrid materials have the needed coherence properties for quantum information processing. Cognitive‐like responses to structured information show a big change in how we see bioquantum hybrid systems. They can now process and remember complex information patterns. A 5‐day exposure to binary‐encoded Shakespearean text showed that proteinoid‐QD networks act like they learn. They adapt gradually in their oscillatory parameters. They also synchronize their frequency with input patterns. Plus, they display memory‐like effects that last even after the stimulation ends. The system can show amplitude modulation responses that match the meaning of the input text. This means the hybrid network can tell apart different types of information. It also adjusts its response, similar to how biological neural networks focus and recognize patterns. This cognitive emergence seems to come from how proteinoid matrices self‐organize and how embedded quantum dots create quantum coherence. Together, they form a computational substrate. This substrate can change its internal structure based on outside influences. The learning rates show exponential adaptation curves. These curves have time constants between 2 and 6 h. They are similar to those seen in biological synaptic plasticity. This suggests that these hybrid systems could act like biological neural networks. They might be able to process, store, and retrieve information at the molecular level.

We have strong proof of quantum‐like coherence and cognitive behavior in proteinoid‐QD systems. But, there are still major gaps in understanding how these effects work. Classical electrochemical models explain basic oscillations and charge transfer. Yet, they miss key quantum effects like entanglement, superposition, and decoherence. These effects are important for grasping the improved phase stability and information processing in hybrid systems. The current theoretical framework isn't advanced enough. It fails to model the quantum‐classical interface. This is where biological self‐assembly processes meet quantum mechanical effects. As a result, there's a gap between what we observe in experiments and what we can predict. Environmental factors like temperature, pH, and ionic strength affect quantum coherence. Yet, we do not fully understand how they work. This lack of knowledge limits our ability to optimize performance for specific applications. Creating better theoretical models is a key challenge. These models must explain the quantum properties of semiconductor nanocrystals. They also need to cover the thermodynamics of protein folding and self‐assembly. Addressing this challenge will move the field from observing to creating and building bioquantum computational systems. Future research should create clear theories linking classical biological physics to quantum mechanics. This will help us model and design proteinoid‐QD networks with specific computational traits. Real‐time quantum state monitoring is key. Techniques like continuous weak measurement and quantum nondemolition measurements let us observe and understand the quantum coherence of these hybrid systems while they operate. Scalability is a big challenge. Moving from lab‐scale tests to real‐world applications needs new ways to build large arrays of proteinoid‐QD networks. These methods must keep the quantum coherence and self‐assembly traits seen in individual units. Use advanced methods like quantum interferometry, coherent control spectroscopy, and single‐molecule tracking. These techniques help explain the tiny mechanisms behind the big behaviors we see. They also help us find the main factors that affect how well the system works. Exploring different quantum dot types, proteinoid sequences, and conjugation methods is key. This will help balance quantum coherence, biological function, and computational performance. In turn, it will lead to bioquantum computing designs. These designs can combine the strengths of biological and quantum systems for advanced information processing.

## 4 Conclusion

This work shows a new approach for bioquantum hybrid materials. It combines Glu‐Phe‐Asp‐Cys proteinoids with semiconductor quantum dots. This integration uses controlled thiol‐maleimide chemistry. We achieved a conjugation efficiency of 80–90%. The networks show properties that go beyond what each part can do alone. They feature unique toroidal self‐assembly designs. SEM analysis revealed a morphological transformation from spherical precursors to toroidal nanostructures with central cavities measuring 300–400 nm in diameter. They also have strong electrochemical oscillations and better signal amplification from quantum effects. The hybrid networks exhibited spontaneous electrochemical oscillations with frequencies ranging from 0.03 to 0.11 Hz and amplitude variations between 297 and 485 mV, reproducible across three independent trials. Incorporation of quantum dots significantly enhanced the signal amplitude, increasing it by a factor of 41 (1999 mV vs. 48.8 mV), likely due to surface plasmon coupling and facilitated charge transfer mechanisms. Electrochemical tests show that the best operating range is 5–20 Hz for real‐world use. Electrochemical impedance spectroscopy (EIS) measurements showed an optimal charge transfer resistance of ≈5250 *Ω*. Square wave voltammetry (SWV) data indicated that electron transfer kinetics follow a first‐order decay with a constant of *α* = 0.0032 Hz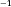. These materials have a 41‐fold increase in amplitude and stable impedance. This makes them great candidates for next‐gen biosensors. They can detect in both optical and electrochemical modes. We see clear oscillatory behavior and frequency synchronization when faced with complex stimuli. The networks responded to structured binary input over a five‐day period, displaying frequency synchronization at *f* = 0.022217 Hz, with magnitude‐squared coherence values of 0.90 for pure proteinoids and 0.85 for proteinoid–QD conjugates. Power spectral density (PSD) analysis showed steep decay slopes ranging from −3.84 to −3.11. Quantum Fisher Information (QFI) calculations revealed changes in coherence sensitivity under different experimental conditions. Future studies should aim to build theories that explain the quantum‐classical link in hybrid materials. They should also look into how to scale these for real‐world computing uses. These findings suggest potential applications in biosensing, bioelectronics, and unconventional computing architectures.

## Conflict of Interest

The authors declare no conflict of interest.

## Data Availability

The data that support the findings of this study are openly available in zenodo at https://zenodo.org/records/17162509, reference number 17162509.
